# A conserved monocyte activation program links brain injury to systemic immune adaptation and clinical outcomes

**DOI:** 10.3389/fimmu.2026.1731068

**Published:** 2026-03-19

**Authors:** Abderrahmane Sadek, Melanie Petrier, Cynthia Fourgeux, Martin Braud, Victor Gourain, Marwan Bouras, Cecile Poulain, Mohieddine Moumni, Antoine Roquilly, Jeremie Poschmann

**Affiliations:** 1 Nantes Université, CHU Nantes, INSERM, Center for Research in Transplantation and Translational Immunology, UMR 1064, Nantes, France; 2 Biotechnology and Bioresources Valorization Laboratory, Biology Department, Faculty of Sciences, Moulay Ismail University, Meknes, Morocco; 3 Service d’Anesthesie Réanimation, Institut de Recherche en Santé 2 Nantes Biotech, Nantes Université, CHU Nantes, Nantes, France

**Keywords:** inflammation, monocytes, transcriptomics, traumatic brain injury, WGCNA (weighted gene co- expression network analyses)

## Abstract

**Rationale:**

Systemic immune dysregulation after traumatic brain injury (TBI) contributes to secondary complications and influences long-term recovery, yet the cellular and molecular dynamics remain incompletely understood.

**Objectives:**

To characterize longitudinal immune responses following TBI and identify conserved transcriptional programs linked to clinical outcomes and mechanistically regulated monocyte states with potential prognostic and therapeutic significance.

**Methods:**

We conducted longitudinal transcriptomic profiling of PBMCs from TBI patients (n =65 samples) at Day 1, Day 7, and Month 6 post-injury, alongside healthy controls (n =24). Cell type composition was assessed using computational deconvolution and validated by flow cytometry. Gene co-expression networks were constructed to identify temporally dynamic immune signatures. Monocyte-specific metabolic activity was inferred using genome-scale metabolic modeling and confirmed by plasma metabolite quantification. Genome-wide chromatin state was assessed by H3K27ac ChIP-sequencing in CD14^+^ monocytes. External validation was performed across independent PBMC and monocyte datasets from bacterial and viral infections, including COVID-19 and pneumonia.

**Main results:**

TBI patients showed a transient increase in circulating monocytes at Day 1 and Day 7, returning to baseline by Month 6. Weighted gene co-expression analysis identified a data-driven monocyte-associated co-expression module (Yellow module) that dominated early responses and tracked with monocyte abundance. This module was conserved in sorted CD14^+^ monocytes from an independent cohort and reflected sustained inflammatory activation. Metabolomic and flux balance analyses revealed coordinated remodeling of the aspartate–arginine–ornithine axis, indicating nitrogen stress and altered metabolic demands during monocyte activation. In external datasets of PBMCs from patients with acute infections, Yellow module expression was associated with clinical severity and independently predicted survival. Epigenomic profiling revealed dynamic enhancer remodeling at Yellow module loci, with PU.1 (SPI1), a monocyte lineage–defining transcription factor, identified as a putative upstream regulator linking transcriptional activation to regulatory architecture.

**Conclusions:**

TBI induces a prognostically informative, monocyte-enriched transcriptional program marked by inflammatory and metabolic reprogramming. This epigenetically regulated state is conserved across critical illness, correlates with patient outcomes, and identifies PU.1 as a key regulator of monocyte activation. These findings support the Yellow module as a candidate biomarker for risk stratification and a mechanistic link between brain injury and systemic immune adaptation.

## Introduction

Traumatic brain injury (TBI) is a significant global health concern, affecting millions of individuals annually and leading to considerable morbidity and mortality (1). Beyond its direct mechanical and vascular effects on the brain parenchyma, TBI triggers a complex neuroinflammatory cascade that involves resident microglia, astrocytes and infiltrating myeloid cells within and around the lesion core and can persist for months or years after the initial insult (2, 3). Traditionally, the focus has been on the immediate neurological damage caused by TBI; however, growing evidence indicates that the injury initiates complex, long-term systemic effects that profoundly influence patient recovery. These brain peripheral interactions include autonomic and endocrine signals that reshape hematopoiesis and circulating leukocyte function and link focal brain damage to systemic immune dysregulation (4, 5). One of the most critical systemic consequences is the alteration of the immune system, which can persist for months post-injury and impact overall health outcomes (6). Importantly, peripheral immune cells do not simply mirror the central nervous system response but can both reflect and modulate tissue level inflammation, making them a clinically accessible window into ongoing brain pathology (7).

Among these systemic immune changes, patients with TBI are notably susceptible to infections and viral reactivations, complications that are associated with worsened neurological outcomes (8). Early after injury, many cohorts report a stereotyped pattern of neutrophilia, lymphopenia and dynamic changes in the neutrophil-to-lymphocyte ratio, and these indices associate with infection risk and functional outcome (4, 9). These immune challenges highlight that TBI’s impact extends beyond the central nervous system, affecting the body’s ability to respond to pathogens effectively. This systemic immune dysfunction suggests that long-term alterations in immune cell function may play a pivotal role in patient recovery and rehabilitation (10). Consistent with this view, experimental and clinical studies indicate that innate and adaptive immune cells show chronic functional changes months after TBI, including impaired cytokine production, altered phagocytosis and features of immunoparalysis (4, 8, 10).

A key aspect of this immune alteration involves circulating monocytes, which are essential for pathogen clearance and immune regulation. We and others have shown that following acute inflammatory events like TBI or systemic inflammatory response syndrome (SIRS), human monocytes exhibit persistent impairments in phagocytic activity (10). These impairments are linked to altered expression of surface receptors and regulators crucial for immune function, including SIRPα, CD206, CD14, and CD163. Such changes result in a shift in monocyte phenotype, leading to a reduced capacity to phagocytize harmful bacteria and respond to pathogens effectively (8). Notably, increased expression of SIRPα in circulating monocytes correlates with the severity of inflammation and a higher susceptibility to infections like pneumonia, underscoring the potential for prolonged immune compromise in TBI patients (10). We previously identified a monocyte-specific transcriptional signature (Brown module) in patients with acute brain injury, defined by weighted gene co-expression network analysis (WGCNA) and labeled “Brown” according to the internal WGCNA color convention, which was characterized by impaired interferon signaling and associated with herpesvirus reactivation and poor neurological outcomes (8). In that study, samples were obtained at a single time point early after injury, so the temporal evolution of this signature and its relationship to later immune states remained unknown. We note that WGCNA module colors are assigned independently in each dataset and do not imply that a Brown module in one cohort is equivalent to a Brown or Yellow module in another. These findings linked suppressed antiviral gene expression to functional immune defects in a clinically relevant population. Importantly, this signature was defined at a single timepoint early after injury, limiting insight into its temporal evolution and broader immunological context.

Given the profound implications for patient outcomes, there is a pressing need to comprehensively understand the temporal dynamics of immune alterations following TBI. Longitudinal profiling can distinguish transient responses from persistent dysregulation and reveal immune trajectories associated with recovery or secondary complications. Longitudinal transcriptomic analyses offer a powerful approach to characterize these immune changes over time, capturing the evolution of gene expression and cellular responses in patients. We have previously shown that both sepsis and sterile inflammation can induce long-lasting immune reprogramming in humans and mice, including persistent phagocytic defects in monocytes and macrophages (10). These alterations can last for months after apparent clinical recovery, affecting susceptibility to infections such as hospital-acquired pneumonia, and also modulating cancer risk through tissue-resident immune reprogramming (11). These findings underscore the need for temporal resolution when studying immune responses to critical illness and motivate the application of longitudinal transcriptomics to TBI. At the same time, they raise the question of whether TBI induces a distinctive monocyte program or instead engages conserved transcriptional states that are shared with other forms of critical illness such as bacterial sepsis and viral pneumonia (5, 12).

In addition to characterizing TBI-specific immune trajectories, an important question is whether the transcriptional programs induced by TBI represent a unique response or are instead part of a broader, conserved pattern of monocyte activation observed across diverse critical illnesses, including bacterial and viral sepsis. Addressing this question could help determine whether monocyte-driven inflammation is a shared pathophysiological feature of severe systemic insults, with implications for generalizable biomarkers and therapeutic strategies.

In this study, we aimed to extend our previous findings by characterizing the immune response to TBI across multiple time points and layers of regulation. We further sought to evaluate the relevance of these transcriptional programs in external cohorts of patients with sepsis and viral pneumonia, using comparable peripheral blood mononuclear cell or monocyte transcriptomes, to test whether the observed monocyte states are specific to brain injury or represent a conserved inflammatory module across critical illness. Establishing such conservation is clinically important because it would support the development of biomarkers and therapeutic targets that are applicable beyond a single disease context and would allow risk stratification based on peripheral blood profiles that can be obtained routinely in intensive care. By identifying specific immune pathways and signatures associated with persistent dysfunction, including those linked to viral reactivation risk and impaired cellular responses, we can gain deeper insights into the long-term consequences of TBI. We initially focused on peripheral blood mononuclear cells (PBMCs) to characterize systemic immune responses to TBI. However, transcriptomic and deconvolution analyses pointed toward a dominant role for monocytes in shaping the observed gene expression changes. We therefore examined their transcriptional, metabolic, and epigenetic remodeling in greater detail. This comprehensive approach allowed us to identify a distinct monocyte inflammatory program, “the Yellow module”, that contrasts with the immunosuppressed phenotype previously defined by the Brown module. By comparing this program across TBI, bacterial sepsis, and viral infections, we aimed to define both disease-specific and shared components of monocyte activation. This knowledge could reveal novel therapeutic targets and inform the optimal timing for interventions aimed at mitigating immune dysfunction, ultimately improving neurological recovery and overall health in TBI patients.

## Results

The study cohort included patients with traumatic brain injury (TBI) and healthy controls to characterize temporal immune alterations after injury. Peripheral blood mononuclear cells (PBMCs) were collected longitudinally from the same TBI patients at three time points: day 1 (D1), day 7 (D7), and month 6 (M6) post-injury. PBMC RNA sequencing was performed on a total of 65 samples, including 20 samples at D1, 23 at D7, and 22 at M6. Due to incomplete longitudinal sampling, not all patients contributed samples at each time point. Among the 65 samples, 12 patients contributed samples at all three time points, while 8 patients contributed samples at two time points and 12 patients contributed a sample at only one time point. A healthy control group (n=24) was used for baseline comparisons. Demographic characteristics were balanced across groups, with mean ages between 42 and 45 years and a predominance of male participants (75–83% among TBI patients and 82% in controls; **Table 1**). Pneumonia was common among TBI patients, with 45–59% of the sampled patients having developed hospital-acquired pneumonia during their intensive care unit (ICU) stay (**Table 1**). At inclusion, patients exhibited marked neurological severity. Median Glasgow Coma Score was 6 at Day 1 and Day 7, with more than half of patients classified as severe traumatic brain injury and most remaining patients classified as moderate traumatic brain injury (**Table 1**). Nearly all patients required intensive care unit admission and invasive mechanical ventilation, indicating a cohort of critically ill, frequently comatose patients at the time of sampling.

**Table 1:** Demographic and clinical characteristics of the study cohort, including age, sex, and relevant clinical features at each time point.

Characteristic	Healthy (n=24)	D1 (n=20)	D7 (n=23)	M6 (n=22)	P value
Demographics					
Age (years), median [IQR]	45.5 [36.0–58.0]	39.0 [35.2–52.8]	39.0 [35.0–64.0]	43.0 [36.8–56.2]	0.54¹
Sex (% male)	71.4	77.3	76.5	77.3	>0.99²
Clinical assessment at admission					
Glasgow Coma Score, median [IQR]	0	6.0 [3.2–9.8]	6.0 [3.0–9.5]	9.0 [6.0–10.8]	<0.0001¹
Glasgow Class, n (%)					<0.0001²
Severe (3-8)	0 (0)	13 (59)	9 (53)	10 (45)	
Moderate (9-12)	0 (0)	9 (41)	8 (47)	11 (50)	
Mild (13-15)	0	0 (0)	0 (0)	1 (5)	
ICU metrics and complications					
ICU admission, n (%)	0 (0)	20 (100)	23(100)	24(95)	<0.0001²
ICU mortality, n (%)	0 (0)	2 (9)	0 (0)	0 (0)	0.20²
ICU length of stay (days), median [IQR]	0 [0-0]	21.5 [15.2-30.0]	24.0 [16.0-35.0]	21.5 [14.8-33.8]	<0.0001¹
Mechanical ventilation (days), median [IQR]	0 [0-0]	15.0 [7.2-25.0]	15.0 [9.0-26.0]	12.5 [8.0-26.0]	<0.0001¹
Pneumonia, n (%)	0 (0)	13 (59)	8 (47)	10 (45)	<0.0001²
Virological status					
HSV serology (% positive)	0 (0)	10 (45)	9 (53)	10 (45)	0.0002²
HSV reactivation (% positive)	0 (0)	4 (18)	5 (29)	6 (27)	0.02²
Outcomes					
90-day mortality, n (%)	0 (0)	2 (9)	0 (0)	0 (0)	0.20²
90-day GOSE, n (%)					<0.0001²
Death	0 (0)	2 (9)	0 (0)	0 (0)	
Vegetative State	0 (0)	1 (5)	0 (0)	0 (0)	
Severe Disability	0 (0)	5 (23)	5 (29)	6 (27)	
Moderate Disability	0 (0)	1 (5)	1 (6)	1 (5)	
Good Recovery	0 (0)	6 (27)	5 (29)	6 (27)	
Healthy	24 (100)	0 (0)	0 (0)	1 (5)	

**Figure 1:**
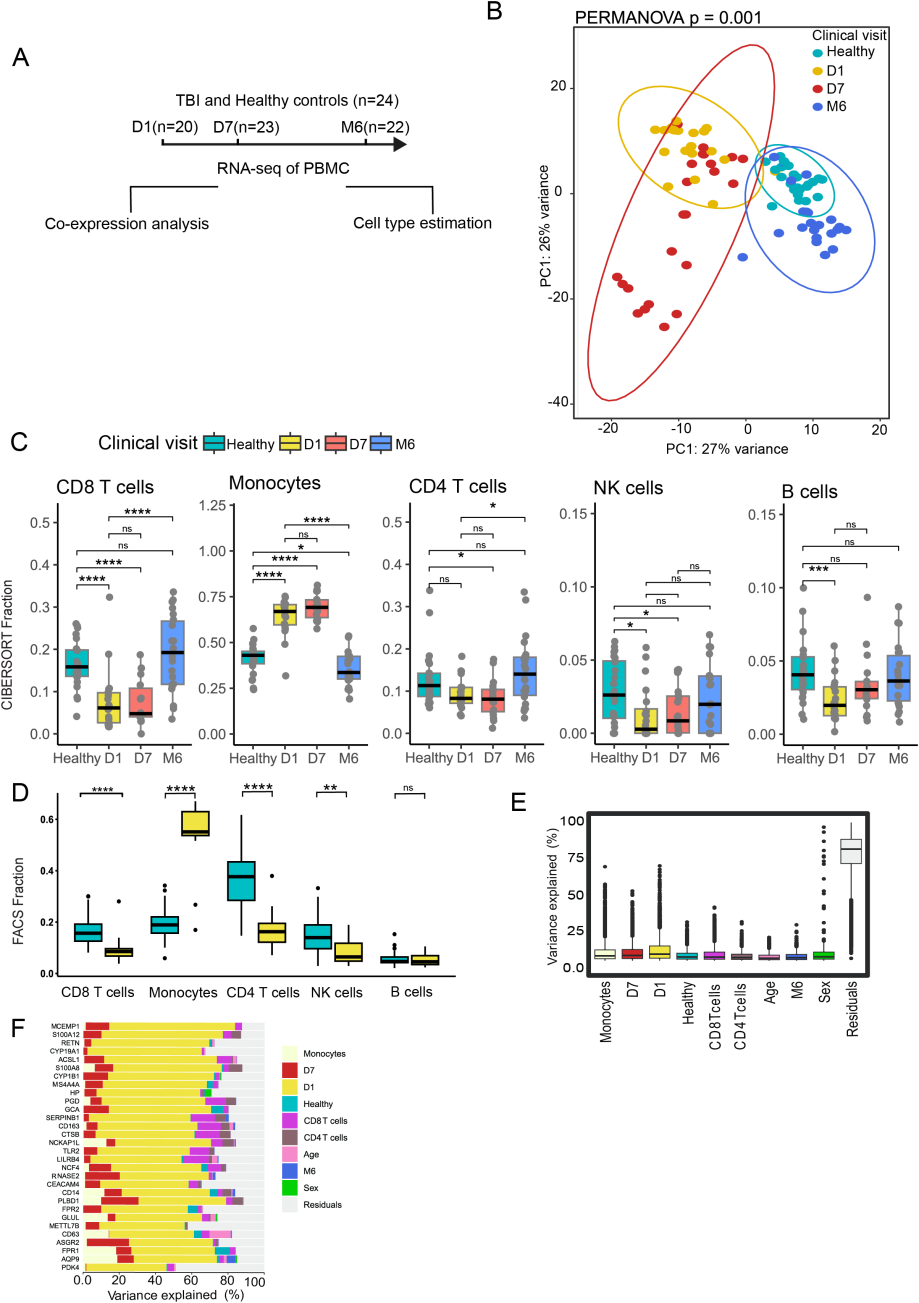
Longitudinal transcriptomic and immune profiling of TBI patients. **(A)** Overview of the analytical workflow, showing RNA sequencing of PBMCs from TBI patients at Day 1 (D1, n = 20), Day 7 (D7, n = 23), and Month 6 (M6, n = 22) after injury and from healthy controls (n = 24), followed by co-expression analysis and immune cell type estimation. B) Principal component analysis (PCA) of transcriptomic profiles, showing separation of samples according to clinical timepoint. Ninety-five percent confidence ellipses are shown for each group. Inter-group differences were quantified using PERMANOVA on Euclidean distances derived from scaled expression data (F = 5.77, R² = 0.178, p = 0.001, 999 permutations). **(C)** Immune cell fraction estimates from CIBERSORTx deconvolution, showing increased monocytes and decreased T cells and NK cells at early time points post-TBI (Kruskal Wallis test followed by Dunn *post hoc* testing with Benjamini Hochberg correction, adjusted p values shown as significance symbols.). Note: Sample sizes differ across time points (D1 = 20, D7 = 23, M6 = 22, Healthy = 24). **(D)** Flow cytometry validation of immune cell proportions, confirming significantly increased monocytes and reduced CD8^+^ T cells at Day 1 compared to healthy controls (P values indicated) (Wilcoxon test with Benjamini–Hochberg FDR correction; adjusted p-values shown as significance symbols). Note: Sample sizes differ across time points (D1 = 11, Healthy =34). **(E)** Variance partitioning analysis showing the proportion of gene expression variance explained by biological and demographic factors. Box plots represent gene-level variance explained by monocyte proportions, time point, health status, T cell subsets, age, and sex; residuals indicate unexplained variance. **(F)** Variance explained for representative genes by each factor. Bars show the percentage of variance attributed to monocytes, clinical time point (D1, D7, M6), health status, T cell subsets, age, sex, and residuals. All analyses were performed using unbiased, data-driven approaches without prior assumptions, allowing the identification of dominant sources of variance and immune cell–associated transcriptional patterns emerging directly from the dataset. Legend key for adjusted p-values: ns = not significant (p ≥ 0.05), * = p < 0.05, ** = p < 0.01, *** = p < 0.001, **** = p < 0.0001.

The overall study design and analysis workflow are summarized in **Figures 1A**, where PBMC samples collected at each time point from TBI patients and healthy controls were subjected to bulk RNA sequencing and used both for gene co-expression network construction and for estimation of immune cell composition.

To capture the systemic immune response to TBI, we performed longitudinal transcriptomic profiling of PBMCs using RNA sequencing. As shown in **Figures 1B**, Principal Component Analysis (PCA) demonstrated a clear temporal structure in the transcriptomic data. PCA revealed a time-associated structure in PBMC transcriptomes, with early time points separated from healthy controls and Month 6 samples along PC1, and a distinction between Healthy and Month 6 along PC2. Permutational multivariate analysis of variance confirmed a significant effect of group on PBMC transcriptomic profiles, explaining 17.8% of the total variance (R² = 0.178, F = 5.77, P = 0.001; **Supplementary Table 1**). This separation indicates that TBI induces marked and dynamic transcriptomic remodeling, with early alterations partially resolving by six months post-injury.

To determine whether the observed variance was attributable to changes in the composition of PBMC immune cell types, we performed cell type deconvolution on the transcriptomic data using CIBERSORT (13), a computational algorithm that infers the relative abundance of immune cell subsets from bulk gene expression profiles (**Figure 1C**). This analysis revealed notable alterations in the proportions of various immune cells in TBI patients compared to healthy controls (**Supplementary Table 1**). There was a significant increase in monocyte fractions at Day 1 and Day 7 post-injury, which returned to levels comparable to healthy controls by Month 6. Conversely, CD4+ and CD8+ T cell proportions decreased at Day 1 and Day 7 but normalized by Month 6.

To validate the computational estimations of immune cell proportions, flow cytometry (FACS) analysis was conducted on PBMC samples from TBI patients on Day 1 and healthy controls (**Supplementary Figures 1D**, **Figure 1A**). The FACS results corroborated the transcriptomic findings, confirming significant differences in the proportions of monocytes and T cells at Day 1 post-injury. These findings reinforce the conclusion that TBI leads to sustained alterations in immune cell composition across time.

To determine whether the observed transcriptional changes were solely due to alterations in cell type composition or also reflected disease-specific effects, we employed the variancePartition framework (14) to decompose the variance in gene expression into its contributing factors (**Supplementary Figures 1E**, **Figure 1B**). This approach utilizes a linear mixed model to quantify the fraction of variance explained by each variable in the study design, including biological factors such as cell type proportions and sampling time points, while correcting for other variables (14). **Figure 1E** therefore summarizes, across all expressed genes, how much variance is attributable on average to monocyte proportion, visit, lymphocyte subsets and demographic factors. Monocyte proportions, together with sampling time points at Day 1 and Day 7, explained the highest amount of variance across the dataset when residual variance was not considered (**Figure 1E**). Consistent with this observation, correlation analysis revealed a significant inverse relationship between the transcriptomic component associated with Day 1 and estimated monocyte fractions (r = –0.44, P = 4.1 × 10^−45;^ **Supplementary Figure 1C**), indicating that samples with stronger Day 1 signatures were enriched for monocyte-driven expression profiles. In comparison, healthy control status, CD8+ T cell proportions, and sex each accounted for a smaller but comparable fraction of variance, whereas CD4+ T cells, age, and Month 6 contributed less. These findings indicate that acute post-injury status and monocyte expansion are the dominant sources of transcriptional variability, while demographic factors exert more modest effects. Importantly, this analysis shows that although monocytes are proportionally more abundant in TBI patients, it is the variance decomposition that specifically distinguishes their contribution to transcriptional remodeling from mere differences in cell frequency.

To further investigate the drivers of gene expression variability, we examined genes with the highest proportion of variance explained in our dataset (**Figure 1F**). For each gene we fitted the same variancePartition model and displayed the percentage of variance attributed to each covariate as a stacked bar, selecting the 30 genes with the largest variance explained by biological variables. This organization means that genes at the top of the panel are those for which visit and monocyte proportion together account for most of the non-residual variance. Several genes were strongly influenced by Day 1 time-point. Notably, S100A8 and S100A12, which encode calcium-binding proteins of the S100 family, showed marked Day 1–driven variance. These molecules act as alarmins, a class of endogenous danger signals released during tissue injury that amplify innate immune activation and promote neutrophil–monocyte crosstalk. Their upregulation highlights the early inflammatory burst characteristic of the acute post-injury phase. Similarly, CD14, a co-receptor for bacterial lipopolysaccharide sensing, also showed Day 1–driven expression variance, further supporting the prominent role of monocytes in the early transcriptomic response to TBI.

Taken together, our results demonstrate that TBI induces significant temporal changes in gene expression and immune cell composition, with monocyte-driven gene expression changes.

### Co-expression network analysis reveals distinct immune and homeostatic gene programs following TBI

**Figure 2:**
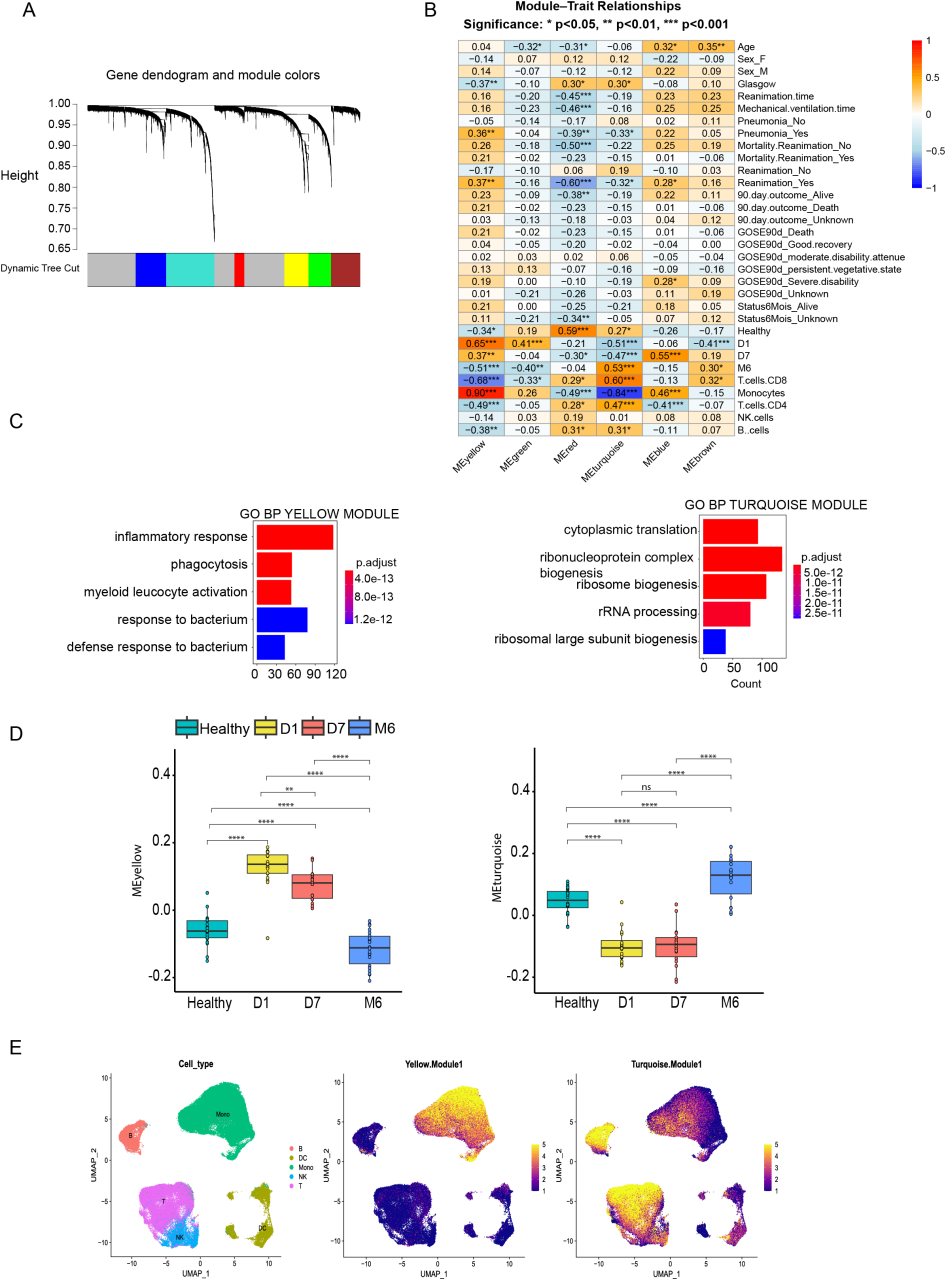
Weighted Gene Co-expression Network Analysis (WGCNA) identifies transcriptional modules and their correlations with clinical and immune parameters. **(A)** Gene dendrogram and module colors generated through hierarchical clustering and dynamic tree cutting in WGCNA. Six co-expression modules were identified, each represented by a distinct color. **(B)** Correlation matrix depicting the relationships between module eigengenes and various clinical and immune parameters. Positive correlations are indicated in red, and negative correlations in blue, illustrating the diverse associations of the six modules with specific biological and clinical features Correlation coefficients were assessed using Pearson correlation. P-values were corrected for multiple testing using the Benjamini–Hochberg procedure; adjusted p-values are shown as significance symbols.). **(C)** Gene Ontology (GO) enrichment analysis for the Yellow and Turquoise modules, highlighting their association with immune responses and protein synthesis pathways, respectively Significance was assessed using a hypergeometric test with multiple testing correction applied to control the false discovery rate (FDR), and a threshold of adjusted p < 0.05 was considered significant. **(D)** Module eigengene (ME) values plotted across time points post-TBI and healthy controls, providing an overview of the temporal expression patterns of the six modules. The temporal dynamics of the Yellow and Turquoise modules are highlighted. Overall group differences were assessed using a Kruskal–Wallis test, followed by Dunn *post hoc* testing with Benjamini–Hochberg FDR correction; adjusted p-values are shown as significance symbols. **(E)** UMAP plots from single-cell RNA-sequencing analysis showing immune cell populations (left) and module-specific gene expression patterns for the Yellow (middle) and Turquoise (right) modules. The Yellow module is predominantly expressed in monocytes, while the Turquoise module shows broader expression across multiple cell types. Legend key for adjusted p-values: ns, not significant (p ≥ 0.05), *= p < 0.05, ** = p < 0.01, *** = p < 0.001, **** = p < 0.0001.

To identify coordinated transcriptional programs rather than individual differentially expressed genes, we used weighted gene co-expression network analysis (WGCNA), an unsupervised approach that groups genes into modules based on shared expression patterns across samples. Each module represents a set of genes whose expression varies in a highly correlated manner across patients and time points. For each module, a single summary value, termed the module eigengene, was computed as the first principal component of the module gene expression matrix and reflects the overall activity of that gene program in each sample. These eigengene values were then used to characterize temporal dynamics and to assess associations with clinical and immune parameters (**Figure 2**; see Methods and **Supplementary Figure 2**) (15). This approach clustered genes into six modules, each representing a distinct co-expression pattern (**Figure 2A**). We next assessed the relationship between each module and clinical or immune parameters using correlation analysis (**Figure 2B**). The Yellow module was consistently associated with greater clinical severity: higher module expression correlated with lower Glasgow Coma Scale scores, as well as with an increased occurrence of pneumonia and a higher need for reanimation (**Figure 2B**). Its expression was higher in samples collected on Day 1 and Day 7 and decreased by Month 6 and in healthy controls. Regarding immune cell composition, the Yellow module was positively correlated with monocyte proportions and negatively correlated with both CD4+ and CD8+ T cell frequencies, suggesting that it captures an early monocyte-driven inflammatory response after traumatic brain injury. In contrast, the Turquoise module displayed significant positive correlations with healthy controls and Month 6 samples and was positively associated with T cell populations, reinforcing the prominent contribution of monocytes to the early post-injury transcriptome.

To clarify the biological significance of the modules, we conducted Gene Ontology (GO) enrichment analysis (**Figure 2C**). The Yellow module was enriched for immune-related processes, such as inflammatory responses, phagocytosis, and myeloid leukocyte activation. The Turquoise module, on the other hand, was associated with processes central to protein synthesis and cellular homeostasis, including cytoplasmic translation, ribosome biogenesis, and rRNA processing. The enriched pathways were consistent with the underlying expression patterns and highlight that the two modules capture distinct aspects of the immune response to TBI.

To further explore how each module’s overall expression, summarized by its eigengene (the first principal component of the module’s gene expression matrix), changed over time, we plotted module eigengene (ME) values across the three TBI time points and healthy controls (**Figure 2D**). The Yellow ME was elevated at Day 1 and Day 7 post-injury relative to healthy controls, mirroring the increased monocyte fraction during these phases and supporting its role in acute inflammatory and immune activation. By Month 6, however, the Yellow ME dropped below control levels, suggesting not only resolution of the early inflammatory response but also a potential shift toward an immune-suppressed or altered homeostatic state.

In contrast to the sustained activation of the Yellow module, the Turquoise module, enriched for ribosomal biogenesis and translational processes, displayed an opposite temporal pattern. Turquoise module eigengene values were reduced at Day 1 and Day 7 compared to healthy controls, consistent with early suppression of fundamental cellular functions. By Month 6, Turquoise eigengene values exceeded those observed in healthy controls, indicating a rebound beyond baseline levels rather than simple normalization.

To validate these co-expression findings and identify the cell types driving module expression, we analyzed a publicly available PBMC single-cell RNA-sequencing dataset from Reyes et al. comprising septic patients and controls profiled at single-cell resolution (16) (**Figure 2E**). Cell types were assigned following the authors’ annotation strategy based on canonical marker genes and clustering. UMAP projection showed clear monocytes, T cells, B cells, dendritic cells, and NK cells. The Yellow module genes were predominantly expressed in monocytes, consistent with their correlation with monocyte proportions, whereas the Turquoise module showed broader expression across multiple immune populations. To assess whether these patterns were preserved across clinical conditions, we additionally visualized module expression using phenotype-split UMAPs. When stratified by disease state, Yellow module expression remained consistently enriched in monocyte clusters across all conditions, while Turquoise module expression retained a more diffuse distribution across immune cell types (**Supplementary Figure 2C**). These results indicate that the cellular specificity of the Yellow and Turquoise modules is robust and independent of the underlying inflammatory context, rather than being driven by a single disease condition.

Together, these findings extend the results of our variance partitioning and cell type deconvolution analyses, offering a systems-level view of coordinated transcriptomic programs during TBI. The Yellow module reflects a monocyte-associated inflammatory response that is strongly induced in the acute phase and falls below baseline by Month 6, consistent with an overshoot of resolution or a shift toward altered immune homeostasis. The Turquoise module instead shows early suppression relative to healthy with recovery by Month 6, mirroring the progressive normalization of core cellular processes. These longitudinal patterns indicate that TBI triggers not only transient immune activation but also longer-term reorganization of peripheral immune programs.

### Yellow module expression in monocytes mirrors acute inflammatory activation across cohorts

**Figure 3:**
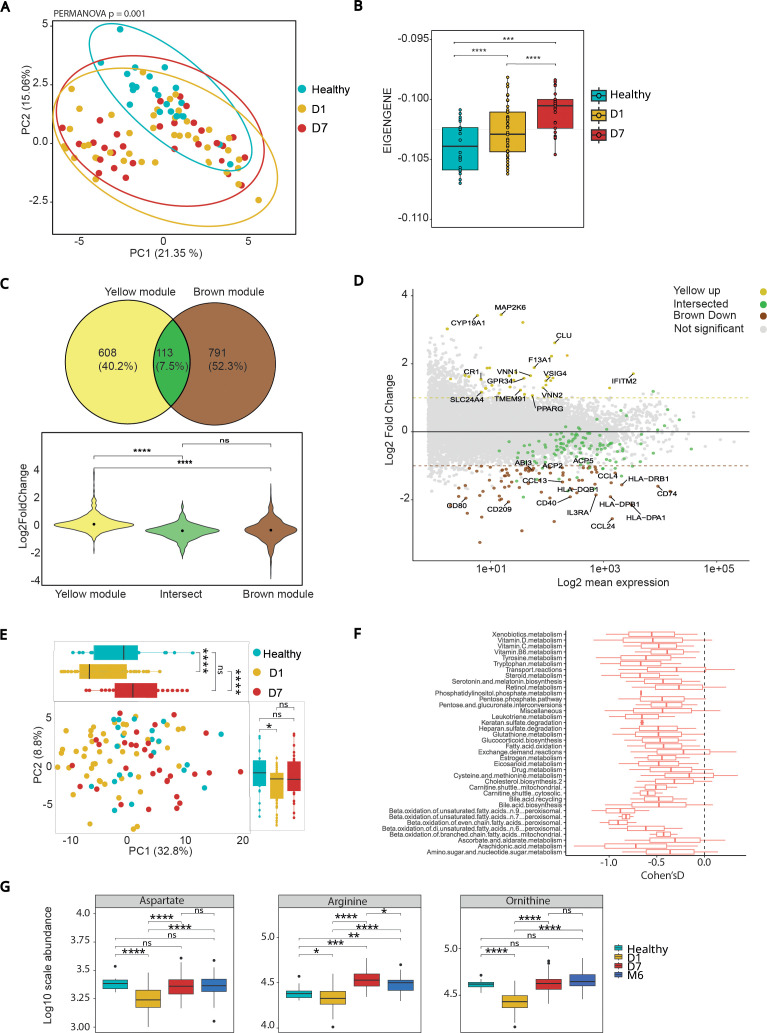
Expression and validation of the Yellow module in CD14^+^ monocytes and comparison with the Brown module. **(A)** Principal component analysis (PCA) of CD14^+^ monocyte transcriptomes from healthy controls, Day 1, and Day 7 TBI patients based on Yellow module gene expression. Ninety-five percent confidence ellipses are shown for each group. Inter-group differences were quantified using PERMANOVA on Euclidean distances derived from scaled expression data (F = 2.13, R² = 0.045, p = 0.001, 999 permutations). **(B)** Yellow module eigengene values plotted by clinical group. Expression is increased at Day 1 and Day 7 post-injury compared to healthy controls, with a further increase from Day 1 to Day 7, indicating persistent activation of Yellow module genes in monocytes. Overall group differences were assessed using a Kruskal–Wallis test, followed by Dunn *post hoc* testing with Benjamini–Hochberg FDR correction; adjusted p-values are shown as significance symbols.Note: Sample sizes differ across time points (D1 = 43, D7 = 29, Healthy = 22). **(C)** Gene overlap and differential expression trends between the Yellow module **(this study)** and the Brown module (Chaumette et al.). The Venn diagram shows limited gene overlap, indicating that the modules capture distinct transcriptional programs. Violin plots display opposing expression trajectories from Day 1 to Day 7, with Yellow genes upregulated and Brown genes downregulated (Wilcoxon test with Benjamini–Hochberg FDR correction; adjusted p-values shown as significance symbols). **(D)** MA plot of differential gene expression (Day 7 vs Day 1) highlighting genes from the Yellow module (yellow), Brown module (brown), and their intersection (grey). Brown genes are predominantly downregulated, while Yellow genes are enriched among upregulated transcripts. Intersecting genes show intermediate behavior, supporting the distinct regulatory profiles of each module. **(E)** PCA of inferred metabolic fluxes in CD14^+^ monocytes from healthy controls, Day 1, and Day 7 TBI patients. Ninety-five percent confidence ellipses are shown for each group. Inter-group differences were quantified using PERMANOVA on Euclidean distances derived from scaled flux values (999 permutations; (F and R² values reported in the Methods)). Separation along PC2 reflects progressive metabolic remodeling following brain injury. **(F)** Pathway enrichment based on Cohen’s D effect size comparing Day 1 TBI patients to healthy controls. Notable alterations include mitochondrial and peroxisomal fatty acid oxidation, bile acid metabolism, and amino acid pathways. Disruptions in nitrogen-handling pathways such as amino sugar metabolism and glutathione metabolism suggest dysfunction of the aspartate–arginine–ornithine axis. **(G)** Plasma concentrations of aspartate, arginine, and ornithine measured at Day 1, Day 7, and Month 6 post-injury. Aspartate levels were decreased acutely and partially recovered by Month 6. Arginine and ornithine showed early depletion followed by rebound at Day 7 and normalization by Month 6. These systemic metabolite trajectories are concordant with pathway-level alterations inferred from monocyte transcriptomes, supporting coordinated remodeling of urea-cycle–related amino acid metabolism during recovery. Statistical comparisons were performed using Wilcoxon tests with Benjamini–Hochberg FDR correction; adjusted p-values are indicated. Legend key for adjusted p-values: ns, not significant (p ≥ 0.05), * = p < 0.05, ** = p < 0.01, *** = p < 0.001, **** = p < 0.0001.

To determine whether the Yellow module identified in PBMCs corresponds to a monocyte intrinsic inflammatory program, we analyzed transcriptomic data from sorted CD14^+^ monocytes obtained from the discovery and validation cohorts described by Chaumette et al. These cohorts include traumatic brain injury (TBI) patients and matched healthy controls, with monocytes collected at day 1 and day 7 post-injury. This independent dataset was used specifically to validate whether the Yellow module identified in our PBMC analysis is preserved in purified monocytes. Expression values for Yellow module genes were extracted and subjected to principal component analysis (PCA) (**Figure 3A**). This analysis revealed a clear separation between healthy controls and TBI patients along PC2, which explained 21% of the total variance. In contrast, Day 1 and Day 7 samples largely overlapped, suggesting minimal temporal separation during early recovery. Healthy controls clustered more tightly, indicating stable expression of Yellow module genes. Consistent with the PCA, permutational multivariate analysis of variance confirmed a significant but modest effect of group on Yellow module expression in CD14^+^ monocytes (PERMANOVA, R² = 0.045, F = 2.13, P = 0.001; Supplementary **Table 1**). These findings confirm that the Yellow module signature is preserved in purified monocytes and primarily distinguishes brain-injured patients from healthy individuals, rather than differentiating acute post-injury time points (**Figure 3A**).

To assess Yellow module expression over time, we compared module eigengene values in CD14^+^ monocytes from the combined Chaumette cohorts (**Figure 3B**). Analyses were performed across all TBI patients irrespective of HSV reactivation status. Expression was significantly higher in TBI patients compared to healthy controls at both Day 1 (P = 4.1 × 10^−5^) and Day 7 (P = 3.7 × 10^−10^), with a modest but significant increase from Day 1 to Day 7 (P = 7.1 × 10^−4^). These findings indicate that the monocyte-associated Yellow module is upregulated early after injury and continues to rise during the first week, supporting a sustained activation pattern in the subacute phase of TBI.

### Yellow and brown modules represent distinct monocyte programs following brain injury

In parallel to the Yellow module identified here, Chaumette et al. previously described a co expression module in sorted CD14^+^ monocytes, referred to as the Brown module, encompassing 721 genes enriched for interferon mediated antiviral responses and antigen presentation pathways, representing a distinct antiviral transcriptional program (8). This module was globally downregulated in patients with brain injury compared to healthy controls and further suppressed in those who developed HSV reactivation, where it was associated with poor neurological recovery at six months. Together, these findings indicate that brain injury induces a broad suppression of monocyte antiviral functions, which contrasts with the inflammatory program captured by the Yellow module.

We next examined the direction and magnitude of gene regulation within each module across time. Violin plots of log_2_ fold change between day 1 and day 7 (**Figure 3C**) revealed opposing expression trajectories: Yellow module genes were predominantly upregulated over time, whereas Brown module genes were largely downregulated. Genes shared between the two modules displayed intermediate changes, consistent with partial regulatory overlap.

These trends were further visualized using an MA plot of differential gene expression (day 7 vs day 1) (**Figure 3D**). Yellow module genes were enriched among transcripts showing increased expression at day 7, while Brown module genes were preferentially represented among downregulated transcripts. Genes belonging to the intersection of both modules exhibited intermediate behavior, reinforcing the conclusion that the Yellow and Brown modules reflect distinct regulatory responses following brain injury. Together, these results demonstrate that the Yellow and Brown modules correspond to separate monocyte transcriptional programs following brain injury. The Brown module reflects a globally dampened interferon-driven antiviral network that is further suppressed in patients with HSV reactivation, whereas the Yellow module defines a concurrent inflammatory program that is broadly induced in the early post-injury phase and increases from Day 1 to Day 7.

### Metabolic characterization of circulating monocytes following TBI

Given that the Yellow module reflected strong activation of monocyte inflammatory programs, we next asked whether these transcriptional changes were accompanied by coordinated alterations in cellular metabolism. To link immune transcriptomic states to metabolic function, we performed a genome-scale flux balance analysis using the METAFlux framework, which infers pathway-level metabolic activity from gene expression profiles by incorporating stoichiometric constraints and gene–reaction associations (17). Importantly, this analysis provides model-based predictions of metabolic fluxes and does not rely on direct metabolite measurements. This approach was applied to CD14^+^ monocyte transcriptomes to identify early metabolic disruptions following brain injury.

Principal component analysis of METAFlux-inferred pathway fluxes revealed a clear separation between TBI patients and healthy controls (**Figure 3E**). PC1, accounting for 32.8% of the variance, distinguished healthy controls from both Day 1 and Day 7 samples (P < 0.0001), while PC2 (8.8% of the variance) showed a modest separation between the two post-injury time points (P = 0.03). This group associated structure was supported by permutational multivariate analysis of variance, which identified a significant effect of clinical group on inferred metabolic flux profiles, explaining 10.5% of the total variance (PERMANOVA, R² = 0.105, F = 5.54, P = 0.001, **Supplementary Table 1**). These results indicate that predicted monocyte metabolic programs are markedly altered after TBI and continue to evolve over the first week. To quantify differences between TBI patients and healthy controls, we performed pathway-level comparisons based on Cohen’s D effect size highlighted extensive metabolic remodeling (**Figure 3F**). Among the most affected pathways were glutathione metabolism, amino sugar and nucleotide sugar metabolism, and several modules involved in fatty-acid oxidation and exchange reactions. Notably, these altered pathways intersect with the urea-cycle–related amino acid metabolism, specifically implicating the aspartate–arginine–ornithine axis. This network links nitrogen handling, redox balance, and intermediary metabolism, suggesting that brain injury triggers a coordinated disturbance of mitochondrial and amino acid fluxes. Several of these pathways converge on nitrogen and redox metabolism and intersect with the urea cycle and amino acid turnover (18, 19).

Based on these model-derived predictions, we next focused on quantifying plasma metabolites directly related to these affected pathways (specifically aspartate, arginine, and ornithine) to assess whether monocyte-inferred metabolic alterations were reflected at the systemic level. Plasma metabolite quantification was performed on plasma samples and not on sorted CD14^+^ monocytes, thereby enabling evaluation of whether cell-intrinsic metabolic reprogramming was accompanied by coordinated changes in the circulating metabolic environment. Plasma levels of these metabolites were measured at multiple time points (D1, D7, M6). Aspartate levels were significantly decreased at D1 and D7 compared to controls, with partial recovery by M6. Arginine concentrations showed an initial reduction at D1, followed by an overshoot at D7 and normalization at M6. Ornithine mirrored this profile, displaying an early depletion with subsequent rebound (**Figure 3G**). Although measured in plasma rather than in isolated monocytes, these trajectories closely mirror the pathway-level alterations predicted by flux modeling of CD14^+^ monocyte transcriptomes, indicating concordance between cell-intrinsic metabolic programs and systemic metabolic readouts. Together, flux predictions and targeted metabolite measurements reveal robust remodeling of the aspartate–arginine–ornithine module after brain injury. This cross-scale agreement strengthens the biological relevance of the inferred metabolic pathways and aligns with clinical data showing reduced circulating arginine metabolites in severe TBI patients (20) and the role of ornithine and arginase pathway activity in macrophage inflammation (21).

### Exploration of yellow module expression across diverse inflammatory and infectious conditions

To evaluate whether the Yellow module represents a conserved transcriptional program across distinct inflammatory settings, we next tested its expression in multiple external datasets. We first analyzed further the dataset from Reyes et al. (16), which performed scRNA-seq of PBMCs from patients spanning a spectrum of bacterial infection severity, ranging from bacteremia without ICU admission to ICU treated sepsis with organ dysfunction. These included patients with bacteremia but no ICU admission (Bac-SEP), patients admitted to the ICU with or without sepsis (ICU-SEP, ICU-NoSEP), and healthy controls.

**Figure 4:**
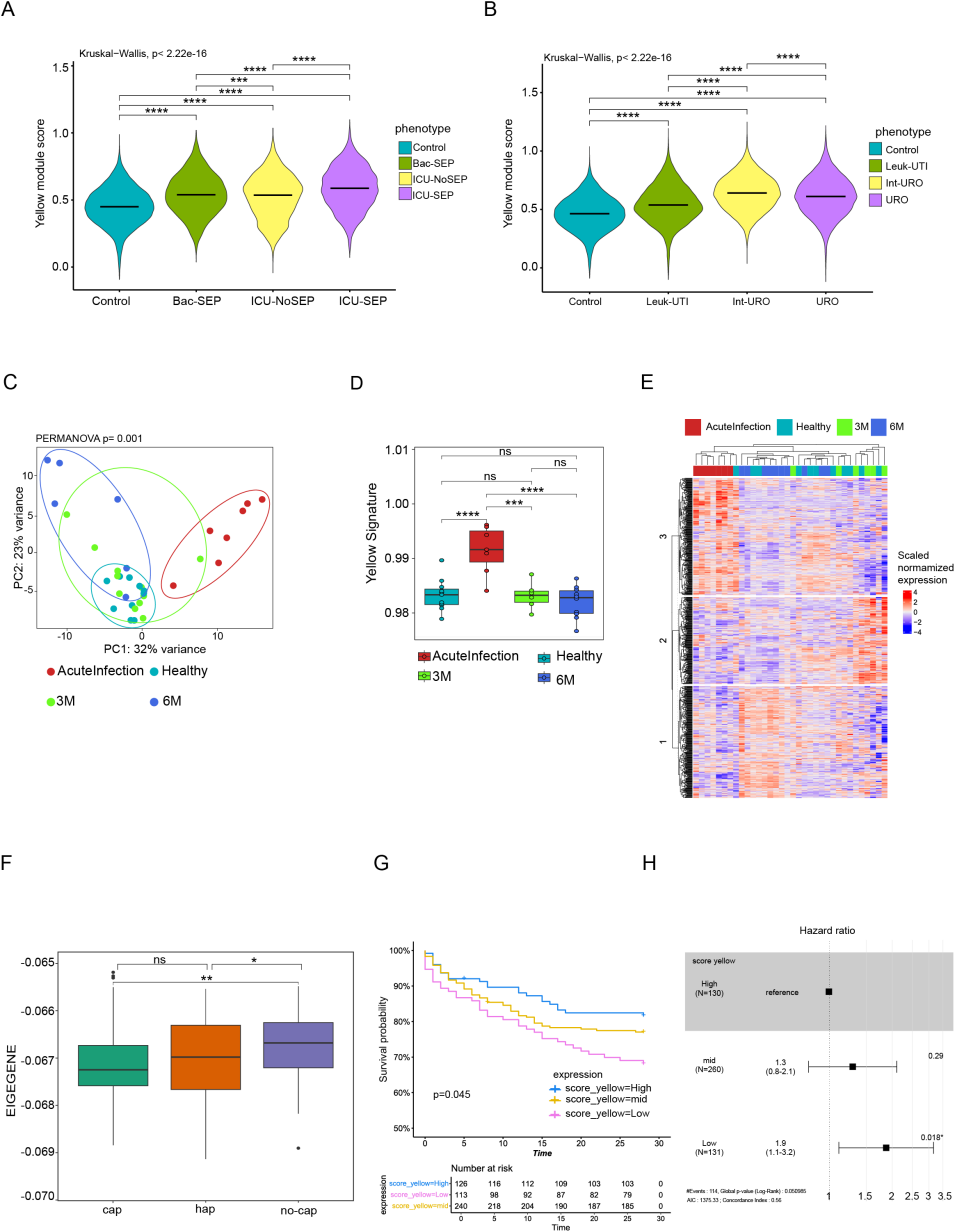
Cross-cohort validation of the Yellow module in diverse inflammatory conditions and viral infection. **(A)** Yellow module scores in PBMC transcriptomes from bacterial sepsis patients across clinical strata in the Reyes et al. dataset, including healthy controls, bacteremic patients without ICU admission (Bac-SEP), ICU patients without sepsis (ICU-NoSEP), and ICU patients with sepsis (ICU-SEP). Overall group differences were assessed using a Kruskal–Wallis test (p < 2.22 × 10^−16^), followed by Dunn *post hoc* testing with Benjamini–Hochberg FDR correction for multiple comparisons. **(B)** Yellow module scores in urinary tract infection phenotypes from the same cohort, including leukocytosis without organ dysfunction (Leuk-UTI), intermediate organ dysfunction (Int-URO), and urosepsis (URO), compared with healthy controls. Overall group differences were assessed using a Kruskal–Wallis test (p < 2.22 × 10^−16^), followed by Dunn *post hoc* testing with Benjamini–Hochberg FDR correction. **(C)** PCA of sorted monocyte transcriptomes from acute COVID-19 patients and individuals sampled at 3 and 6 months post-recovery (Brauns et al.). Ninety-five percent confidence ellipses are shown for each group. Inter-group differences were quantified using PERMANOVA on Euclidean distances derived from scaled expression data (F = 7.53, R² = 0.043, p = 0.001, 999 permutations). Sample sizes differ across time points (Healthy = 10, Acute = 7, M3 = 11, M6 = 6). **(D)** Yellow module scores at acute, 3-month, and 6-month time points in the COVID-19 cohort Overall group differences were assessed using a Kruskal–Wallis test, followed by Dunn *post hoc* testing with Benjamini–Hochberg FDR correction; adjusted p-values are shown as significance symbols. **(E)** Heatmap of scaled Yellow module gene expression across disease states showing three temporal gene clusters. **(F)** Yellow module eigengene values across clinical subgroups in the MARS cohort, including community-acquired pneumonia (CAP), hospital-acquired pneumonia (HAP), and non-infectious controls (no-CAP). Overall group differences were assessed using a Kruskal–Wallis test, followed by Dunn *post hoc* testing with Benjamini–Hochberg FDR correction. **(G)** Kaplan–Meier survival analysis of ICU patients stratified by Yellow module expression tertiles. **(H)** Cox proportional-hazards model evaluating risk of death by Yellow module expression level. ns = not significant (p ≥ 0.05), * = p < 0.05, ** = p < 0.01, *** = p < 0.001.

Yellow module scores, computed in the single cell transcriptomes, were significantly elevated in all patient groups relative to controls (**Figure 4A**). Notably, the highest scores were observed in ICU-SEP patients, followed by Bac-SEP and ICU-NoSEP. This progressive increase aligned with clinical severity, indicating that Yellow module expression scales with the intensity of systemic immune activation. While pairwise comparisons were highly significant across groups, the primary information conveyed by these analyses is the graded shift in Yellow module expression across increasing levels of clinical severity rather than the absolute magnitude of individual pairwise differences. To further examine the relationship between Yellow module expression and clinical severity, we conducted a focused analysis using the primary cohort from Reyes et al., who stratified patients with urinary tract infection (UTI) based on organ dysfunction severity. This included patients with leukocytosis but no organ dysfunction (Leuk-UTI), those with mild or transient dysfunction (Int-URO), and those with sustained urosepsis (URO), alongside healthy controls. Yellow module scores increased progressively with severity across these phenotypes (**Figure 4B**). Compared to controls, all disease groups showed significantly elevated module expression. Notably, expression was higher in Int-URO compared to Leuk-UTI, and highest in URO patients, consistent with a dose–response relationship between Yellow module activation and the extent of systemic organ dysfunction. This supports the interpretation that the Yellow module captures a graded inflammatory program in monocytes that correlates with infection severity.

To evaluate whether the Yellow module captures conserved monocyte states beyond bacterial infections, we analyzed transcriptomic data from sorted monocytes of COVID-19 patients from the study by Brauns et al. (22). This dataset included individuals sampled during acute severe SARS-CoV-2 infection as well as at 3- and 6-months post-recovery, alongside healthy controls. First, we verified that the yellow module was conserved in this dataset (**Supplementary Figure 4A**). PCA based on yellow module expression revealed clear separation between acute and convalescent phases, with acute samples forming a distinct cluster from healthy and recovered individuals (**Figure 4C**). This separation was supported by permutational multivariate analysis of variance, which identified a significant effect of clinical group on Yellow module expression in monocytes (PERMANOVA, R² = 0.043, F = 7.53, P = 0.001). Yellow module scores were significantly elevated during acute infection (P = 7.5 × 10^−4^ vs. healthy), followed by a stepwise reduction at 3 months and further normalization by 6 months (**Figure 4D**). No significant difference was observed between 6-month recovery and control samples, indicating resolution of monocyte activation over time. Hierarchical clustering of Yellow module genes (**Figure 4E**) revealed a temporally ordered structure rather than a uniform activation pattern. Genes within cluster 1 were predominantly upregulated during the acute phase, whereas cluster 2 genes showed higher expression at 3 months, and cluster 3 genes were enriched at 6 months. This indicates that distinct subsets of Yellow module genes are engaged at different stages of the immune response and recovery, reflecting a gradual shift in monocyte transcriptional programs over time. These findings are in line with the original report by Brauns et al., which demonstrated a transient but profound alteration in monocyte differentiation and function in severe COVID-19 (22). The dynamic behavior of the Yellow module across this trajectory supports its utility as a marker of monocyte activation in acute viral inflammation, and its progressive decline aligns with immunological recovery.

To investigate whether Yellow module expression holds clinical relevance in predicting outcomes during critical illness, we analyzed transcriptional data from the MARS consortium study (23), which includes genome-wide blood expression profiles from ICU patients with community-acquired pneumonia (CAP), hospital-acquired pneumonia (HAP), and non-infectious causes of critical illness (no-CAP). RNA was collected at ICU admission, and patients were followed longitudinally for survival outcomes. We first confirmed that the Yellow module was conserved in this dataset (**Supplementary Figure 4B**). Given that the Yellow module tracked monocyte activation across multiple infectious contexts and scaled with clinical severity, we next examined whether its expression also stratified survival outcomes within this severely ill population. Yellow module eigengene expression differed significantly across clinical categories (**Figure 4F**). In contrast to our initial hypothesis, eigengene values were lower in both CAP and HAP patients compared to the non-infectious (no-CAP) group (CAP vs no-CAP, P = 0.0064; HAP vs no-CAP, P = 0.034). The similar reduction in CAP and HAP suggests that module downregulation is a shared feature of infectious critical illness rather than specific to infection source or site.

Next, we stratified patients into tertiles based on their Yellow module eigengene values and evaluated survival using Kaplan–Meier survival analysis. Because lower eigengene values were associated with infection-related states, we tested whether this downregulation predicted worse outcomes. Patients with low Yellow module expression exhibited significantly lower survival probability compared to those with high expression (log-rank P = 0.045; **Figure 4G**). Cox proportional hazards modeling confirmed this association, showing that low expression was associated with a 1.9-fold increased hazard of death (95% CI: 1.1–3.2; P = 0.018) relative to the high-expression group (**Figure 4H**). The mid-expression group showed an intermediate, non-significant trend. Importantly, survival did not differ between patients with community-acquired (CAP) and hospital-acquired (HAP) pneumonia (log-rank P = 0.67; **Supplementary Figure 4C**), indicating that the association between low Yellow module expression and poor outcome is independent of infection origin. This association should be interpreted as reflecting the link between impaired monocyte activation and adverse clinical trajectories in severe infection, rather than as evidence of a causal or severity independent effect on survival.

Together, these findings suggest that suppression of the Yellow module reflects monocyte dysfunction in severe infection and is linked to adverse clinical trajectories. Integrating data from Reyes et al., Brauns et al., and the MARS cohort, the Yellow module thus emerges as a conserved, stimulus-responsive monocyte program whose dysregulation scales with disease severity and survival risk in critical illness.

### H3K27ac profiling reveals temporal epigenomic remodeling in monocytes near yellow module genes in critically Ill COVID-19 patients

To determine whether the transcriptional program captured by the Yellow module is supported by coordinated chromatin-level regulation, we next examined epigenomic changes in monocytes during severe viral critical illness. Because the Yellow module was originally identified in TBI and validated in independent bacterial and viral datasets, we focused on a longitudinal cohort of critically ill COVID-19 patients as an additional model of systemic inflammation.

This epigenomic dataset was generated independently of the TBI cohort and consists of ICU COVID-19 patients sampled longitudinally, providing an opportunity to assess whether the same monocyte program identified transcriptionally is also reflected at the level of enhancer activity.

**Figure 5:**
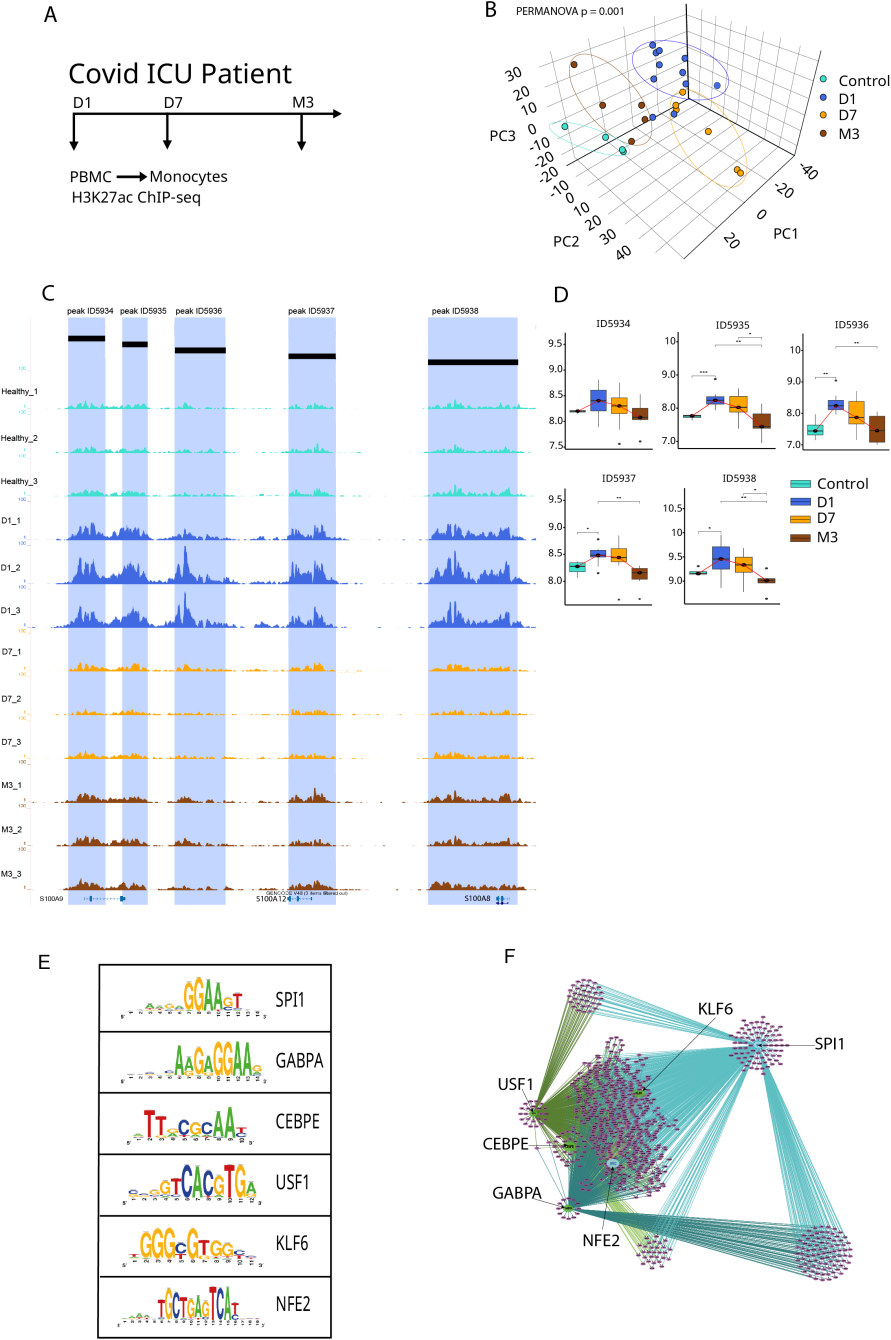
Epigenomic remodeling of CD14^+^ monocytes at Yellow module genes in critically ill COVID-19 patients. **(A)** Study design of an independent longitudinal cohort of critically ill COVID-19 patients, showing PBMC sampling at Day 1 (D1), Day 7 (D7), and Month 3 (M3) for H3K27ac ChIP-sequencing in CD14^+^ monocytes, alongside healthy volunteer controls. **(B)** Principal component analysis (PCA) of H3K27ac signal at regulatory regions linked to Yellow module genes, summarizing chromatin activity across disease stages. Ninety-five percent confidence ellipses are shown for each group. Inter-group differences were quantified using PERMANOVA on Euclidean distances derived from scaled peak intensity data (F = 1.99, R² = 0.33, p = 0.001, 999 permutations). Sample sizes differ across time points (Control = 4, D1 = 11, D7 = 5, M3 = 5). **(C)** Representative genome browser tracks of the S100A8–S100A12 locus in CD14^+^ monocytes showing five distinct H3K27ac peaks with differential acetylation across timepoints. **(D)** Quantification of H3K27ac signal at the five peaks shown in panel C, illustrating coordinated temporal changes in enhancer activity. Overall differences in peak intensities were assessed using one-way ANOVA or Kruskal–Wallis tests depending on distributional assumptions, followed by appropriate *post hoc* testing with Benjamini–Hochberg correction, as detailed in the Statistical Analysis and Supplementary Table. **(E)** Motif enrichment analysis of H3K27ac-marked regulatory regions proximal to Yellow module genes, identifying transcription factors associated with the Yellow module regulatory architecture. **(F)** Co-acetylation network of Yellow module–associated enhancers, highlighting a PU.1-centered regulatory structure. Legend key for adjusted p-values: ns, not significant (p ≥ 0.05), * = p < 0.05, ** = p < 0.01, *** = p < 0.001.

We performed H3K27ac ChIP sequencing on CD14^+^ monocytes isolated from peripheral blood at Day 1, Day 7, and Month 3 after ICU admission, together with healthy volunteer controls (**Figure 5A**). H3K27ac marks active enhancers and promoters and therefore provides a readout of regulatory regions engaged in gene transcription (24, 25).

Using H3K27ac peaks linked to Yellow module genes, we observed clear temporal organization of chromatin states across disease stages. Principal component analysis showed separation between acute disease, recovery phases, and healthy controls, indicating dynamic remodeling of regulatory regions associated with the Yellow module (**Figure 5B**). This structure was supported by permutational multivariate analysis of variance, which identified disease stage as a significant source of epigenomic variation (**Supplementary Table**).

To illustrate these changes at individual loci, we examined the S100A8–S100A12 region, which encodes key inflammatory mediators expressed by activated monocytes. Genome browser visualization revealed five discrete H3K27ac peaks at this locus, all of which showed coordinated changes in acetylation over time (**Figures 5C, D**).

Notably, these genes were also among the most strongly upregulated transcriptionally in both TBI and COVID-19 datasets, linking enhancer remodeling to the transcriptional activation captured by the Yellow module. Together, these observations indicate that the Yellow module corresponds to a coherent regulatory program supported by dynamic enhancer activation rather than isolated gene expression changes.

To identify candidate regulators underlying this enhancer program, we performed transcription factor motif enrichment analysis in H3K27ac-marked regions proximal to Yellow module genes. Compared to genome-wide acetylated regions, these enhancers were significantly enriched for motifs corresponding to transcription factors involved in monocyte differentiation and activation, including PU.1, GABPA, CEBPE, USF1, KLF6, and NFE2 (**Figure 5E**).

Network analysis of co-acetylated regions revealed extensive connectivity among these enhancer elements, with PU.1-associated regions forming a central hub linking multiple regulatory nodes (**Figure 5F**).

Overall, these results show that the monocyte activation program initially identified transcriptionally in TBI is underpinned by coordinated epigenomic remodeling in an independent cohort of critically ill COVID-19 patients. This supports the existence of a conserved, PU.1-centered regulatory architecture linking chromatin state, gene expression, and clinical phenotypes across diverse forms of critical illness.

## Discussion

This study provides a comprehensive longitudinal characterization of peripheral immune responses following traumatic brain injury (TBI), with a specific focus on monocyte-related alterations. Our findings reveal two distinct but interconnected phenotypes: an acute and transient increase in circulating monocytes, and a robust transcriptional reprogramming within these cells. Together, these features define a monocyte-driven immune axis that dominates the early post-injury response.

Using deconvolution and flow cytometry, we observed a significant rise in monocyte proportions at Day 1 and Day 7 post-injury, returning toward baseline by Month 6. Variance partitioning confirmed that this expansion was a major contributor to transcriptional variance, but it also revealed that timepoint-specific effects independently contributed to gene expression changes. This indicates that TBI not only alters immune cell composition but also induces monocyte-intrinsic gene expression programs beyond shifts in abundance.

Co-expression network analysis identified the Yellow module as the principal transcriptional signature associated with these early post-TBI monocytes. This module was enriched for inflammatory and phagocytic pathways and closely tracked both monocyte proportions and early post-injury timepoints. Yellow module expression was consistently enriched in monocytes, as confirmed by both scRNA-seq and bulk RNA-seq analyses, including validation in sorted CD14^+^ monocytes from an independent cohort. These results support the presence of a coordinated monocyte transcriptional program that is upregulated acutely after injury and only partially resolves by Month 6.

Comparative analysis with the previously defined Brown module from Chaumette et al. underscores the distinct nature of the Yellow module (8). While the Brown module reflects an interferon-based antiviral program that is suppressed in association with viral reactivation, the Yellow module marks a separate inflammatory state characterized by sustained activation rather than loss of function. The minimal overlap between modules and their opposing temporal dynamics reinforce the concept of divergent monocyte phenotypes following brain injury.

Beyond transcriptional activation, our analysis also identified metabolic remodeling in monocytes following TBI. Genome-scale metabolic modeling and targeted metabolomics indicated early disruption of the aspartate–arginine–ornithine axis, suggesting that nitrogen handling and amino acid metabolism are altered in parallel with immune activation. These findings imply that TBI-associated monocyte activation is coupled to specific metabolic demands, potentially contributing to systemic metabolic stress in the acute phase.

In addition to transcriptional and metabolic findings, our epigenomic analysis revealed that the Yellow module is embedded within a coordinated enhancer landscape characterized by differential H3K27ac marking at key immune loci. Motif enrichment and network analyses identified SPI1 (PU.1) as a central node within this regulatory program. PU.1-associated enhancer regions showed the highest degree of connectivity with other transcription factor–linked sites, indicating that PU.1 may function as a master regulator of Yellow module gene expression. Our epigenomic analysis highlights PU.1 as a central regulatory node within the Yellow module, suggesting a mechanistic basis for monocyte activation that may be amenable to pharmacological modulation (26).

Finally, we demonstrated that the Yellow module is not unique to TBI but is activated in other inflammatory states, including bacterial infections, COVID-19, and pneumonia. Across multiple inflammatory conditions, including an independent ICU cohort, higher Yellow module expression was associated with disease severity and improved survival, supporting its relevance as a generalizable and prognostic marker of monocyte activation. Importantly, in these infectious critical illness cohorts, Yellow module expression should be interpreted as a readout of monocyte functional state that covaries with overall disease severity and clinical trajectory, rather than as an independent or causal determinant of survival.

The epigenomic analyses in critically ill COVID-19 patients were explored to address a mechanistic question rather than to introduce a new disease focus. Specifically, they test whether the monocyte transcriptional program defined by the Yellow module in TBI and validated across infectious cohorts is supported by coordinated chromatin-level regulation in an independent model of systemic inflammation. The observation that Yellow module genes are associated with dynamic H3K27ac remodeling and a conserved PU.1-centered enhancer network indicates that this program reflects a stable regulatory architecture rather than a context-specific transcriptional artifact. Thus, the COVID-19 epigenomic data strengthen the biological interpretation of the Yellow module as a conserved monocyte activation state across forms of critical illness, rather than to shift the scope of the study away from TBI.

This study has several limitations. First, all patients were recruited from a single European clinical center, which may limit the generalizability of the findings across populations with different genetic backgrounds, healthcare systems, or injury patterns. However, validation of key findings was performed in publicly available datasets from diverse inflammatory conditions, supporting the broader relevance of the identified monocyte signature. Second, while we performed in-depth transcriptomic, metabolic, and epigenomic analyses, other layers of molecular regulation such as proteomics, chromatin accessibility (for example, ATAC-seq), and DNA methylation were not explored and may reveal additional regulatory mechanisms. Third, our analyses focused primarily on monocytes, given their dominant contribution to early transcriptional variance and their specific enrichment for the Yellow module. However, other immune and non-immune cell types, including neutrophils, dendritic cells, and endothelial cells, may also play significant roles in the systemic response to brain injury and warrant further investigation. This study was conducted in a cohort of patients with predominantly moderate to severe traumatic brain injury requiring intensive care, with most patients presenting with low Glasgow Coma Scores and prolonged mechanical ventilation, and therefore reflects immune trajectories in a population with high initial neurological severity. As a result, these findings may not be directly generalizable to patients with mild traumatic brain injury, who represent the majority of TBI cases. Finally, functional validation of candidate transcriptional regulators such as PU.1 was not performed *in vivo* or ex vivo, and future work will be needed to assess their causal role and therapeutic potential in modulating post-injury immune responses.

### Clinical relevance

The Yellow module represents a robust monocyte activation program that is induced early after traumatic brain injury and persists into the subacute and chronic phases. Biologically, this module is enriched for genes involved in inflammation, phagocytosis, and tissue remodeling—functions that may contribute to the containment of damage, clearance of debris, and promotion of repair processes following injury. These findings suggest that sustained activation of the Yellow module may reflect a protective immune response, and insufficient activation could contribute to adverse outcomes.

Clinically, the expression level of the Yellow module has prognostic value. The outcome associations described here arise from a population of patients with predominantly severe neurocritical illness, characterized by low admission Glasgow Coma Scores, frequent coma, and prolonged intensive care support, which defines the clinical context in which this prognostic signal was identified. Across multiple inflammatory conditions, including an independent ICU cohort, higher Yellow module expression was associated with disease severity and survival, supporting its relevance as a generalizable and prognostic marker of monocyte activation. This suggests that transcriptional profiling of monocytes, or even peripheral blood signatures reflecting Yellow module activity, could aid in risk stratification and outcome prediction in TBI and potentially other forms of critical illness.

From a therapeutic perspective, our epigenomic analyses identify PU.1 (SPI1) as a central transcriptional regulator of the Yellow module. PU.1 integrates multiple upstream signals and coordinates enhancer activity across inflammatory genes. Notably, PU.1 is pharmacologically targetable, and recent small-molecule inhibitors have been shown to modulate its activity in preclinical models (26). These findings raise the possibility that tuning PU.1 function could allow controlled modulation of monocyte activation states, potentially enhancing protective responses while limiting deleterious inflammation.

Additional clinical implications include the potential use of the Yellow module as a biomarker for monitoring immune trajectories after brain injury, guiding patient stratification for clinical trials, or identifying individuals at risk of immune exhaustion or secondary infections. Its consistent activation across diverse inflammatory states also suggests it may serve as a generalizable indicator of monocyte responsiveness, with relevance beyond neurotrauma, including sepsis, pneumonia, and viral infections.

## Methods

### Patient cohort and ethics approvals

Patients with moderate-to-severe traumatic brain injury (TBI) were prospectively enrolled at the University Hospital of Nantes (CHU Nantes, France) between March 2016 and March 2019. Inclusion criteria included Glasgow Coma Scale (GCS) ≤13 at admission and evidence of traumatic brain injury on brain imaging. Patients were predominantly affected by moderate to severe traumatic brain injury at inclusion. Injury severity was assessed using the Glasgow Coma Score at ICU admission and categorized according to standard definitions. The majority of patients had a Glasgow Coma Score below 9, consistent with severe TBI, and required invasive mechanical ventilation and intensive care management.

Exclusion criteria were pre-existing immunosuppression, hematological malignancy, or withdrawal of care within the first 24 hours. Healthy volunteers matched for age and sex were included as controls. Blood samples were collected at Day 1, Day 7, and Month 6 post-injury. Written informed consent was obtained from patients or legal representatives in accordance with the Declaration of Helsinki. The collection of human samples has been declared to the French Ministry of Health (Programme de recherche “Immunologie”, DC-2017-2987), and was approved by the Comite de Protection des Personnes Ouest IV (7/04/2015 and 08/10/2020).

### Covid-19 Cohort

The samples were included in the Immunology Program 13, one of the CHU de Nantes research programs declared to the French Ministry of Health. Patients and or their legal representatives provided written informed consent approved by the local ethics committee Comité de Protection des Personnes on October 8 2020, and the processing of associated data was declared to the French data protection authority CNIL.

### RNA sequencing and transcriptomic profiling

PBMCs were isolated and sorted by fluorescence-activated cell sorting (FACS) to enrich specific immune populations. Total RNA was extracted using the RNeasy Plus Mini Kit (Qiagen). RNA integrity was assessed using the Caliper GX LabChIP nucleic acid analyzer. For transcriptome profiling, libraries were prepared from 10 ng of total RNA using a 3′ digital gene expression protocol described in (8, 27). After template-switching reverse transcription, cDNA molecules were barcoded and amplified. Libraries were tagmented using a transposase-based fragmentation method enriched for the 3′ ends of transcripts. Sequencing was performed on a NovaSeq 6000 platform (Illumina), generating an average of 5 million 75 bp single-end reads per sample. Reads were demultiplexed and aligned to the hg19 reference genome using the DGE pipeline. Low abundance genes (fewer than 10 counts in at least five samples) were filtered prior to downstream analyses.

### Cell type deconvolution and validation

Normalized count matrices were obtained from bulk RNA sequencing data using the Relative Log Expression (RLE) method as implemented in the *DESeq2* R package. These normalized matrices served as input for immune cell-type deconvolution using the CIBERSORT algorithm with 1,000 permutations, accessed via the *IOBR* package. To evaluate the accuracy of cell-type estimation, we compared CIBERSORT-derived proportions with matched flow cytometry (FACS) data by computing Pearson correlations. Bland-Altman analysis was conducted to assess agreement between these modalities.

### Flow cytometry analysis of peripheral blood mononuclear cells

For surface staining of PBMCs, 1–2 × 10^6^ cells were incubated with a viability dye (e.g., Live/Dead Aqua, Thermo Fisher) for 15 minutes at 4 °C, followed by incubation with a panel of fluorochrome-conjugated antibodies targeting lineage and activation markers for 30 minutes at 4 °C in the dark. Antibodies included anti-CD3, CD4, CD8, CD14, CD16, CD19, CD56, HLA-DR, PD-L1, CD80, and corresponding isotype controls. Samples were acquired on a BD LSRFortessa X-20 flow cytometer using FACSDiva software (BD Biosciences). Compensation was performed using single-stained UltraComp eBeads. Data were analyzed in FlowJo v10.8. Gating strategies were defined on FSC/SSC profiles and doublet exclusion, followed by sequential gating to identify major immune populations. Median fluorescence intensity (MFI) and frequency of marker-positive cells were used for quantitative comparisons. Between-group comparisons were performed using Mann-Whitney U tests.

### Gene co-expression network

Filtered count data were variance-stabilized using the *vst* function from the *DESeq2* package. The resulting matrix was used as input to construct weighted gene co-expression networks using the *WGCNA* R package. Signed networks were built with the blockwiseModules function, using the following parameters: TOMType = “signed”, power = 10, mergeCutHeight = 0.15, minModuleSize = 150, maxBlockSize = 12000. Modules were defined from hierarchical clustering of topological overlap matrices.

### Module–trait associations

We assessed associations between module eigengenes and clinical and immunological traits. These included sampling timepoints (Day 1, Day 7, and Month 6 post-injury), Glasgow Coma Scale (GCS) at admission, and estimated immune cell abundances obtained from CIBERSORT (monocytes, T cells CD8, T cells CD4, NK cells, B cells). Additionally, we incorporated herpes simplex virus (HSV) reactivation status recorded during ICU stay, categorized as “No reactivation,” “Unknown reactivation,” or “Post reactivation,” reflecting whether patients showed no evidence of HSV reactivation, indeterminate status, or documented reactivation during follow-up, respectively. These categories were derived from systematic viral PCR screening as previously described (Chaumette et al.).

Pearson correlations were computed between module eigengenes and traits.

### Gene Ontology Enrichment analysis

First gene symbols corresponding to module members were mapped to Entrez Gene identifiers using the org.Hs.eg.db annotation package (V 3.22.0), with unmapped entries excluded. A gene universe was defined from all expressed genes in the dataset to provide an appropriate background for enrichment testing. Gene Ontology (GO) enrichment analysis was performed using the clusterProfiler package (28) (V 4.18.2), focusing on the Biological Process (BP) ontology. Significance was assessed using a hypergeometric test with multiple testing correction applied to control the false discovery rate (FDR), and a threshold of adjusted p < 0.05 was considered significant. Enrichment results were converted to human-readable gene symbols and visualized using the enrichplot package (V 1.30.4), with barplots to highlight the most significantly enriched biological processes. Complete gene ontology results are found in the **Supplementary Table**.

### Module preservation analysis

Preservation of co-expression modules was assessed using the *modulePreservation* function from *WGCNA*. We computed both the Z-summary statistic and the median rank of preservation to evaluate intra-modular connectivity and density conservation across datasets. Z-summary <2 indicated no preservation; values between 2 and 10 indicated weak to moderate preservation; values ≥10 indicated strong preservation. Median rank comparisons accounted for differences in module size.

### Metabolic Flux analysis

We estimated metabolic pathway activity in monocytes at Day 1 and Day 7 using the *METAFlux* R package. The method applies metabolic flux balance analysis to transcriptomic data. Pathway-level activity scores were computed as the mean of flux values across reactions associated with each pathway, based on transcriptomic constraints (17).

### Metabolomic analysis by mass spectrometry

Metabolomic profiling of sorted CD14^+^ monocytes was performed using mass spectrometry. Sample preparation and data acquisition were carried out externally using an untargeted or semi-targeted platform. Metabolite identification and quantification were based on mass-to-charge ratios and retention times, and results were reported as normalized intensities. Analyses focused on central metabolic pathways and selected immunometabolic intermediates.

### Survival analysis and data visualization

Kaplan-Meier curves and Cox regression models were computed using the *survival* package in R. PCA plots and boxplots were generated using *ggplot2*, and clustered heatmaps using *ComplexHeatmap*. Statistical analyses including correlation tests and group comparisons (T-tests or Wilcoxon) were performed using base R or relevant statistical packages.

Generation of metabolome data *(carried out by general metabolomics).*

### Sample preparation

The human serum was stored at -80 °C prior to analysis. Samples were thawed on ice. 20 µL of sample was transferred to a 96-deepwell plate. 180 µL of a chilled solution of 80% methanol in water (v/v) was added to the sample. The plates were capped with a silicone cap mat and mixed with a plate shaker for 15 seconds. The capped plates were incubated for 1 hour at 4°C. The plates were centrifuged for 30 minutes at 3,750 rpm at 4°C. 100 µL of the supernatants were transferred to new 96-well plates and diluted 1:10 in a solution of 80% methanol in water (v/v) for instrument analysis. A pooled study sample (pSS) was prepared by pooling 5 µL of each sample together.

### Instrumental analysis

Metabolome profiles of the sample extracts were acquired using flow-injection mass spectrometry. The method described here is adapted from Fuhrer et al., 2011. The instrumentation consisted of an Agilent 6550 iFunnel LC-MS Q-TOF mass spectrometer in tandem with an MPS3 autosampler (Gerstel) and an Agilent 1260 Infinity II quaternary pump. The running buffer was 60% isopropanol in water (v/v) buffered with 1 mM ammonium fluoride. Hexakis (1H, 1H, 3H-tetrafluoropropoxy)-phosphazene) (Agilent) and 3-amino-1-propanesulfonic acid (HOT) (Sigma Aldrich) were added to the running buffer to serve as lockmasses. The isocratic flow rate was set to 0.150 mL/min. The instrument was run in 4GHz High Resolution, negative ionization mode. Mass spectra between 50 and 1,000 m/z were collected in profile mode. 5 µL of each sample were injected twice, consecutively, within 0.96 minutes to serve as technical replicates. The pooled study sample was injected periodically throughout the batch. Samples were acquired randomly within plates.

### Data processing and annotation

Raw profile data were centroided, merged, and recalibrated using MATLAB software described by Fuhrer et al., 2011. Putative annotations were generated based on compounds contained in the Human Metabolome Database, KEGG, and ChEBI databases using both accurate mass per charge (tolerance 0.001 m/z) and isotopic correlation patterns.

### Analysis of publicly available datasets

To assess the generalizability of the Yellow module and its relevance across diverse inflammatory contexts, we analyzed several publicly available transcriptomic datasets:

1. Reyes et al. (*Nat Med*, 2020) single-cell RNA sequencing of PBMCs from patients with urinary tract infections, stratified by sepsis status and organ dysfunction severity (GEO: GSE167363).
2. Brauns et al. (*JCI Insight*, 2022) RNA sequencing of sorted CD14^+^ monocytes from patients with acute and convalescent COVID-19 (GEO: GSE169687).
3. MARS consortium dataset (*AJRCCM*, 2015) bulk whole-blood transcriptomes from ICU patients with community-acquired pneumonia (CAP), hospital-acquired pneumonia (HAP), or non-infectious critical illness (ArrayExpress: E-MTAB-4421).
4. Chaumette et al. (*AJRCCM*, 2022) transcriptomic profiles of sorted CD14^+^ monocytes from acute brain injury patients and healthy controls (GEO: GSE185555).

All datasets were downloaded from GEO or ArrayExpress and processed using standardized pipelines. Raw count matrices or normalized expression data were imported into R. For single-cell datasets, Seurat (v4.0) was used for filtering, normalization, and dimensionality reduction. For bulk RNA-seq, if raw counts were available, they were normalized using DESeq2 or edgeR. Module eigengenes or gene set scores were computed using the *moduleEigengenes* function from WGCNA or the *AddModuleScore* function from Seurat, based on the Yellow module gene set defined in our primary cohort.

Comparative analyses across disease states and timepoints were performed using nonparametric tests (Wilcoxon or Kruskal-Wallis) for module scores. For survival analysis in the MARS dataset, patients were stratified by tertiles of Yellow module eigengene expression, and outcomes were assessed using Kaplan–Meier curves and Cox proportional hazards models. PCA and hierarchical clustering were used for visualization of module dynamics. When required, gene orthology was confirmed using Ensembl gene IDs or biomaRt to ensure consistency across platforms and species.

### Chromatin immunoprecipitation sequencing

To investigate epigenetic regenrich CD14+ormed H3K27ac ChIP-seq. Cells were sorted by FACS to enrich for CD14^+^ monocytes as previously described here (25). Chromatin was prepared using standard protocols, and chromatin immunoprecipitation was carried out using an anti-H3K27ac antibody (Abcam, clone ab4729). DNA libraries were generated using the NEBNext Ultra II DNA Library Prep Kit (New England Biolabs) and sequenced on a NovaSeq 6000 system (Illumina), yielding 45,000–50,000 peaks per sample.

Peak calling was performed using Dfilter, and peaks present in all samples were retained to build a consensus peak set. Read counts in each peak were quantified using *featureCounts* and normalized. Low-signal peaks were excluded from downstream analysis. Differential acetylation analysis was performed using the *DESeq2* package, adjusting for library size and using the Wald test. Peaks with an adjusted p-value <0.05 and |log_2_ fold-change| >1 were considered differentially acetylated. To annotate peaks, the closest gene was assigned based on transcription start site proximity (± 1 kb). Enrichment for transcription factor binding motifs within differentially acetylated regions was assessed using the Hypergeometric Optimization of Motif EnRichment (HOMER) framework. To identify transcriptional regulators associated with selected co-expression modules, we applied *iRegulon* within Cytoscape on the genes comprising the yellow module. Transcription factor-target enrichment and motif discovery were conducted using default scoring thresholds. Networks were visualized and annotated using Cytoscape.

### Data availability

All data generated in this study are publicly available on Zenodo under the following record: https://zenodo.org/records/18008608. This repository contains the processed RNA seq and ChIP seq data, associated sample metadata, and clinical annotations required to reproduce the analyses presented in the manuscript. The Zenodo record serves as the sole data repository for this study and no additional accession numbers are pending. The Zenodo record includes a gene level RNA seq count matrix for PBMC samples counts_IBIS_PBMC.csv; sample level RNA seq metadata linking individuals, time points, and experimental conditions Meta_IBIS_PBMC.xlsx and mixedmeta_ibis2_deb.csv; processed ChIP seq peak calls in BED format after filtering quantification_DFilter.bed; derived monocyte specific datasets used for downstream analyses vsdMixed_monocytes_IBIS_Deba.csv; and a clinical annotation table for the CovARDS cohort ClinicalData_CovARDS_3.csv. Together, these files correspond to all datasets used in the figures and statistical analyses reported in the manuscript.

### Statistical analysis

PCA PERMANOVA: To formally assess group level differences in the multivariate datasets visualized by principal component analysis, we performed permutational multivariate analysis of variance (PERMANOVA). For each analysis, distance matrices were computed using Euclidean distances on scaled data matrices. Group effects were tested using the adonis2 function from the vegan R package under a reduced model with marginal effects and 999 permutations. PERMANOVA was applied to PBMC transcriptomic profiles, CD14^+^ monocyte transcriptomes, external monocyte transcriptomic datasets, and H3K27ac ChIP sequencing peak intensities. Effect sizes were reported as the proportion of variance explained (R²) together with F statistics and permutation based P values. Ninety five percent confidence ellipses were overlaid on PCA plots for visualization purposes.

With For comparisons across more than two groups, we used an assumption guided approach. Normality was assessed within each group using the Shapiro Wilk test, and homogeneity of variance was assessed using the Levene test with Brown Forsythe as a robustness check. If normality and homoscedasticity assumptions were satisfied, we applied one way ANOVA followed by Tukey HSD for *post hoc* testing. If assumptions were not satisfied, we applied Kruskal Wallis followed by Dunn *post hoc* testing. For all families of *post hoc* tests, p values were adjusted using Benjamini Hochberg false discovery rate control. Two group comparisons used Student t tests when assumptions were met or Wilcoxon rank sum tests otherwise. The results of assumption testing and the full set of *post hoc* comparisons are provided in the **Supplementary Statistical Table**.

## Data Availability

All data generated in this study are publicly available on Zenodo under the following record: https://zenodo.org/records/18008608. This repository contains the processed RNA seq and ChIP seq data, associated sample metadata, and clinical annotations required to reproduce the analyses presented in the manuscript. The Zenodo record serves as the sole data repository for this study and no additional accession numbers are pending.The Zenodo record includes a gene level RNA seq count matrix for PBMC samples counts_IBIS_PBMC.csv; sample level RNA seq metadata linking individuals, time points, and experimental conditions Meta_IBIS_PBMC.xlsx and mixedmeta_ibis2_deb.csv; processed ChIP seq peak calls in BED format after filtering quantification_DFilter.bed; derived monocyte specific datasets used for downstream analyses vsdMixed_monocytes_IBIS_Deba.csv; and a clinical annotation table for the CovARDS cohort ClinicalData_CovARDS_3.csv. Together, these files correspond to all datasets used in the figures and statistical analyses reported in the manuscript.
