# Supplementary material for: A conserved monocyte activation program links brain injury to systemic immune adaptation and clinical outcomes
Source: Front Immunol. 2026 Mar 19;17:1731068. doi: 10.3389/fimmu.2026.1731068 (PMC13043442; doi:10.3389/fimmu.2026.1731068)

Supplementary Figure S1A. Gating strategy for flow cytometric identification of peripheral blood immune subsets from total events. Flow cytometry data were analyzed using a sequential hierarchical gating approach to distinguish myeloid and lymphoid lineages. All events were initially plotted on FSC-A versus SSC-A to segregate lymphoid (low FSC-A/SSC-A) from myeloid (higher FSC-A/SSC-A) populations based on cell size and internal complexity. Doublets were excluded within each lineage by selecting singlet cells on FSC-A versus FSC-H plots. Viable leukocytes were then identified by negative selection of the viability dye among singlets. Within the myeloid gate, viable singlets were resolved into total monocytes ($CD14^+$ events encompassing classical, intermediate, and non-classical subsets) using CD14 versus CD16 expression within the indicated polygonal gate. Parallel analysis of the lymphoid compartment gated viable singlets on CD3 versus CD19 to delineate T cells ($CD3^+CD19^-$) and B cells ($CD3^-CD19^+$); $CD3^+$ events were subdivided into $CD4^+$ and $CD8^+$ T cell subsets based on differential CD4 versus CD8 expression. Natural killer (NK) cells were defined as $CD3^-CD56^+CD16^+$ events among remaining lymphoid singlets. Representative pseudocolor dot plots from a single donor illustrate each gating step. This standardized strategy was applied uniformly across all samples analyzed to quantify absolute frequencies and proportions of monocytes, B cells, $CD4^+$ T cells, $CD8^+$ T cells, and NK cells, as detailed in the Results section.

Supplementary Figure S1B. Variance partitioning of yellow module genes by clinical/compositional covariates using variancePartition. Box plot of standardized Beta coefficients from linear mixed models decomposing per-gene expression variance. Boxs show Beta per gene-covariate (Age, D1, D7, Healthy, monocytes, $CD4^+$ T, $CD8^+$ T cells).

Supplementary Figure S1C. Scatter plot Correlation between Beta coefficients of Day 1 (D1) and Beta coefficients of monocytes for yellow module genes. Each point represents one gene; The linear regression line (red) highlights the strong inverse relationship.

Supplementary Figure S2A. Analysis of network topology to select the soft-thresholding power for the adjacency matrix construction. Numbers within boxes denote the soft-thresholding powers corresponding to squared correlation coefficients (R^2 values).

Supplementary Figure S2B. Sample dendrogram and trait heatmap for WGCNA module detection.

Supplementary Figure S2C. Phenotype-stratified single-cell Uniform manifold (UMAP) approximation and projection of Yellow and Turquoise modules, in the Reyes et al. dataset, separately for each clinical phenotype (e.g., healthy controls, bacteremic patients without

ICU admission (Bac-SEP), ICU patients without sepsis (ICU-NoSEP), and ICU patients with sepsis (ICU-SEP).) to visualize Yellow and Turquoise modules across immune cell clusters.

Supplementary Figure S4A. Module preservation statistics in the GSE198256 dataset.

Left: MedianRank vs. module size; values near zero indicate high preservation.

Right: Zsummary vs. module size; dashed blue/green lines mark thresholds Z = 2 (moderate) and Z = 10 (strong) preservation, respectively. Modules exceeding cutoffs demonstrate robust cross-dataset stability.

Supplementary Figure S4B. Module preservation statistics in the GSE65682 dataset.

Left: MedianRank vs. module size; values near zero indicate high preservation.

Right: Zsummary vs. module size; dashed blue/green lines mark thresholds Z = 2 (moderate) and Z = 10 (strong) preservation, respectively. Modules exceeding cutoffs demonstrate robust cross-dataset stability.

Supplementary Figure S4C. Kaplan–Meier survival analysis of ICU patients stratified by Yellow module expression for hospital-acquired pneumonia (HAP) versus Community-acquired pneumonia (CAP).

Survival curves (blue: HAP; red: CAP) show no significant difference (log-rank P = 0.67). Number at risk tabulated below for 0, 10, 20, and 30 days: HAP (n=106, 94, 85, 0); VVP (n=77, 68, 61, 0).

Supplementary figure 5. Hierarchical clustering heatmap of normalized H3K27ac signal across Yellow module-associated peaks confirms distinct and persistent epigenomic profiles between patients and controls across all timepoints.

supplemental figure 1

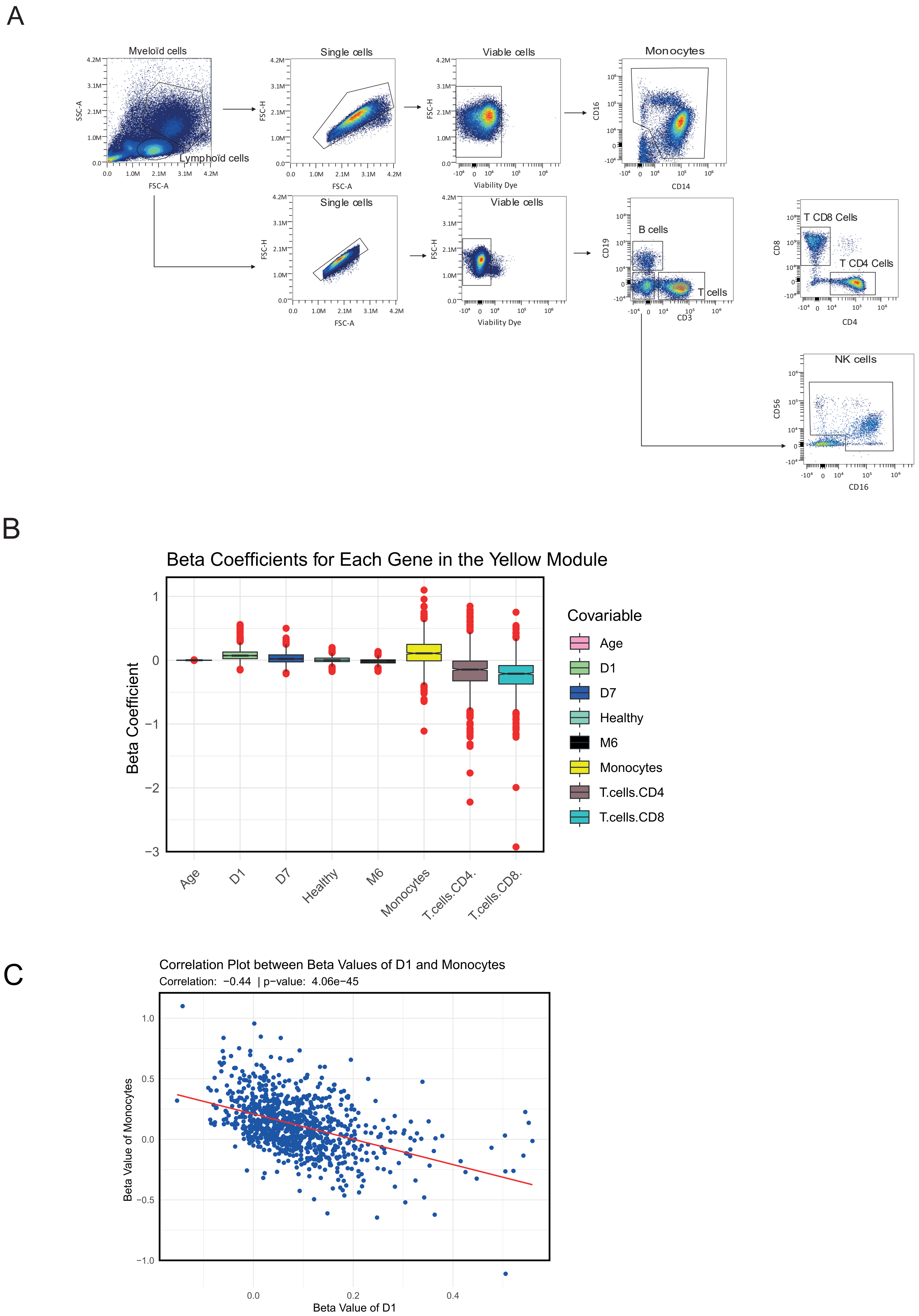

supplemental figure 2

A

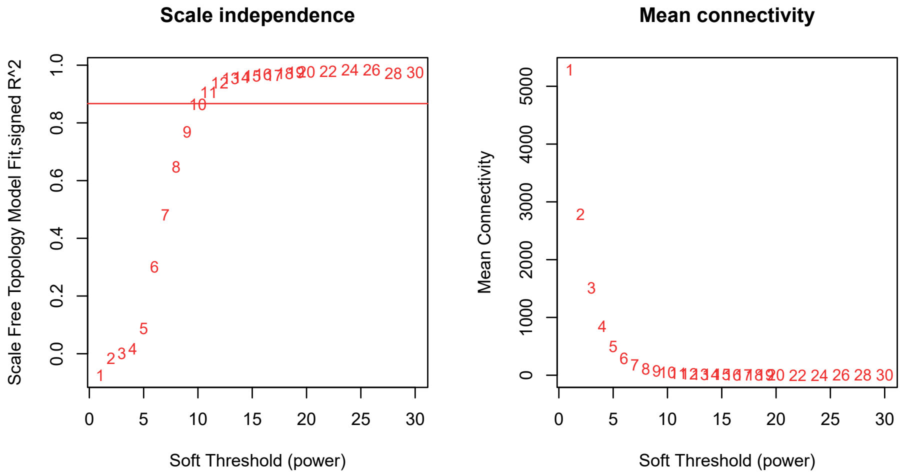

B

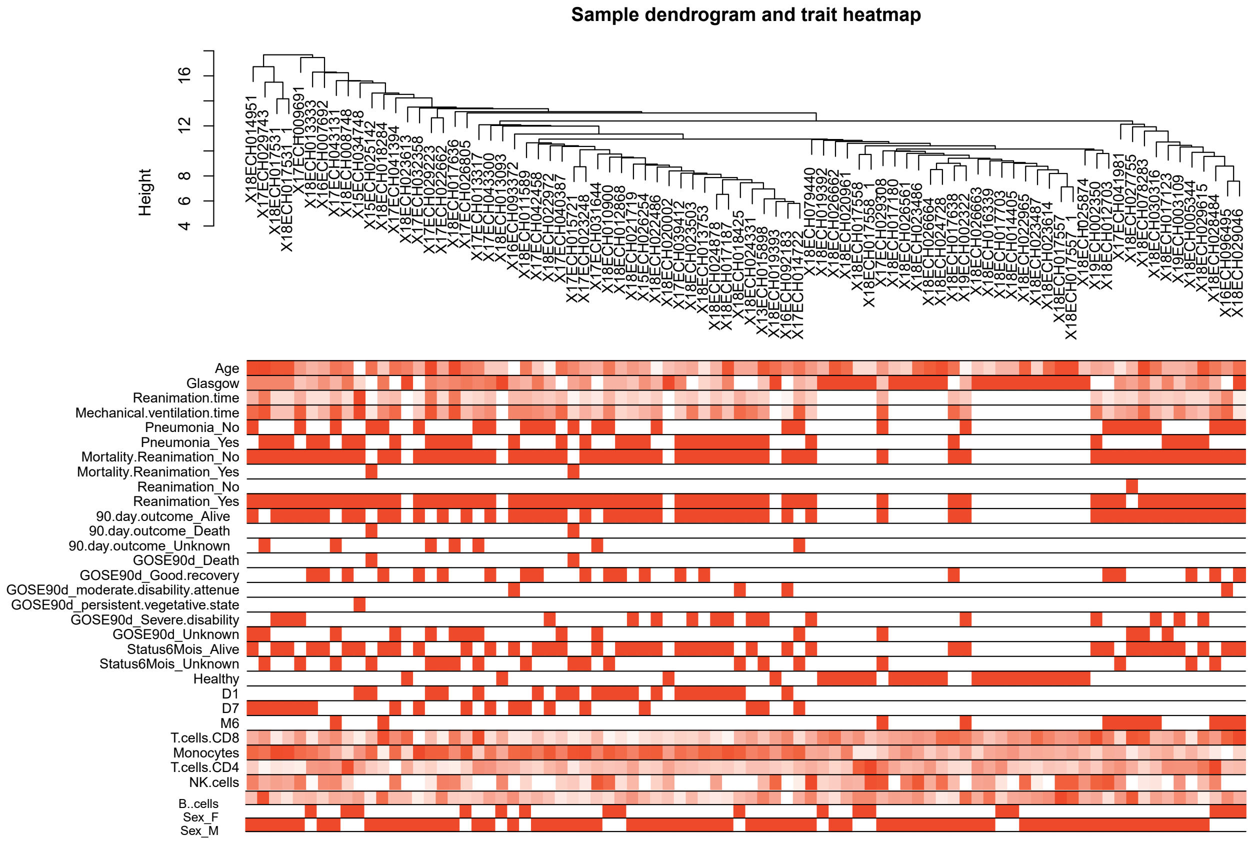

supplemental figure 2

C

yellow module

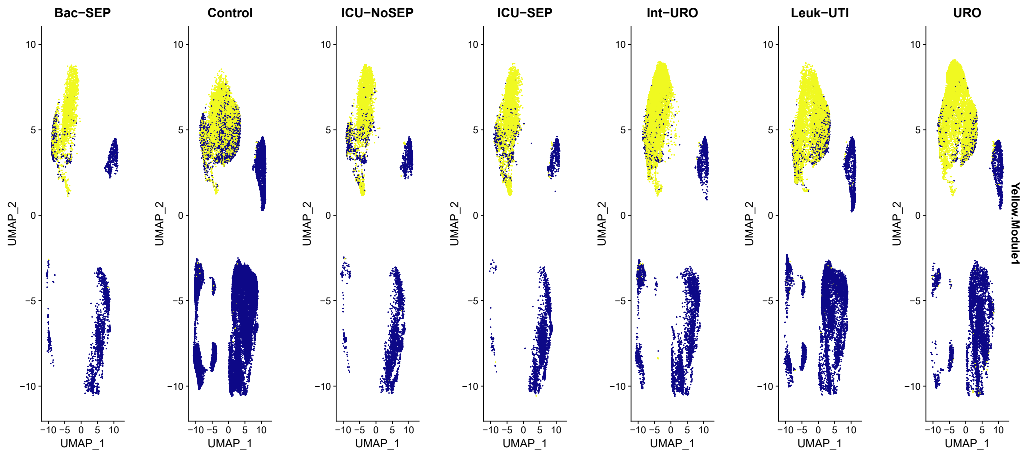

turquoise module

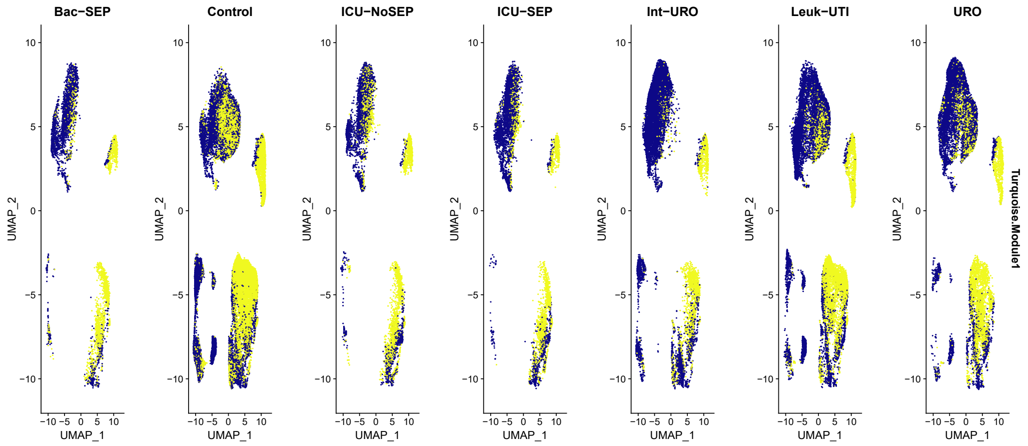

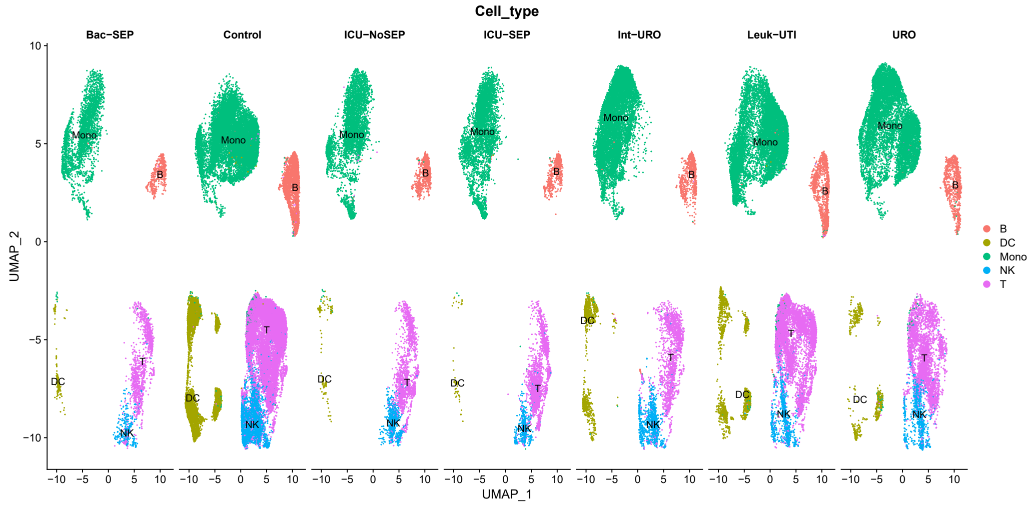

supplemental figure 4

A

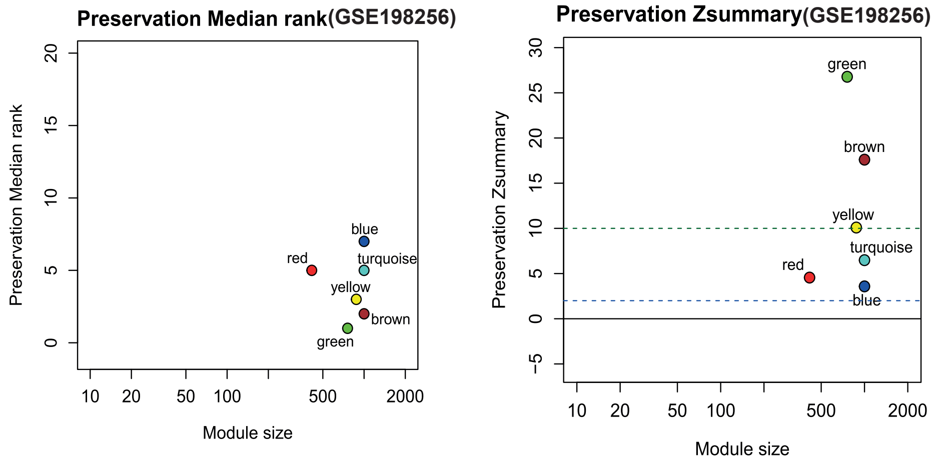

B

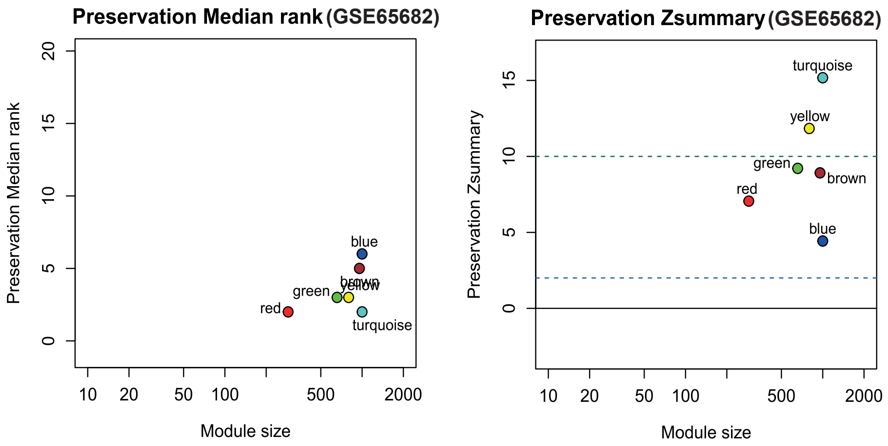

C

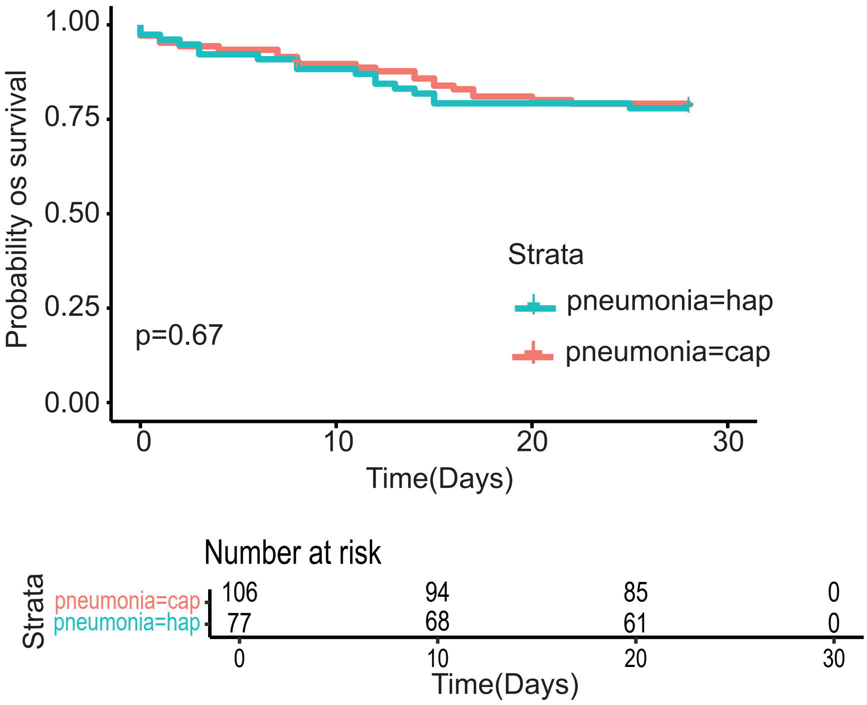

supplemental figure 5

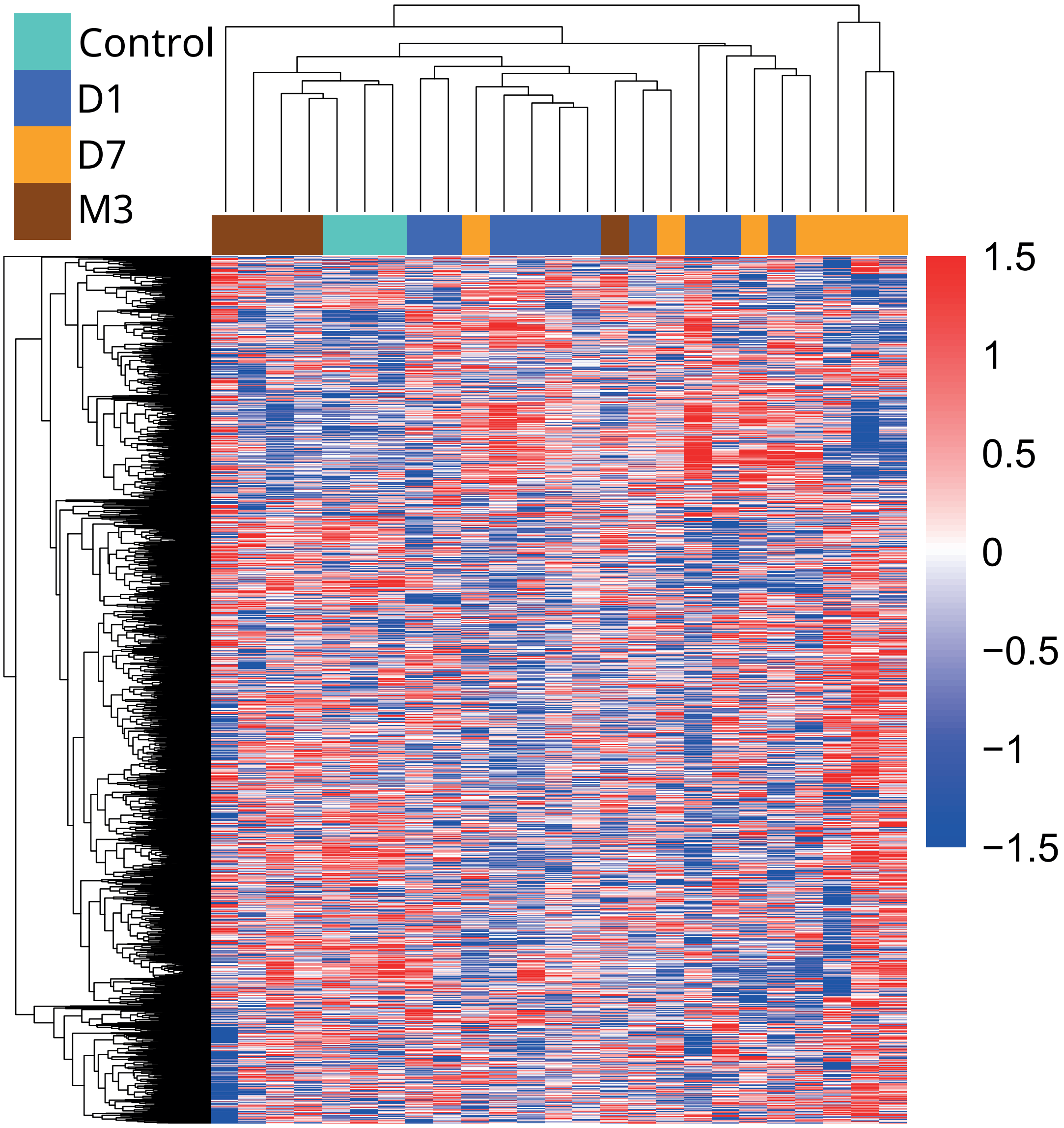

Supplement: Supplementary file 1 [file DataSheet1.pdf]
